# Supplementary material for: Dynamic Palmitoylation of the Sodium-Calcium Exchanger Modulates Its Structure, Affinity for Lipid-Ordered Domains, and Inhibition by XIP
Source: Cell Rep. 2020 Jun 9;31(10):107697. doi: 10.1016/j.celrep.2020.107697 (PMC7296346; doi:10.1016/j.celrep.2020.107697)
Supplement: Document S1. Figure S1 and Table S2 [file mmc1.pdf]

Cell Reports, Volume *31*

Supplemental Information

Dynamic Palmitoylation of the Sodium-Calcium Exchanger Modulates Its Structure, Affinity for Lipid-Ordered Domains, and Inhibition by XIP

Caglar Gök, Fiona Plain, Alan D. Robertson, Jacqueline Howie, George S. Baillie, Niall J. Fraser, and William Fuller

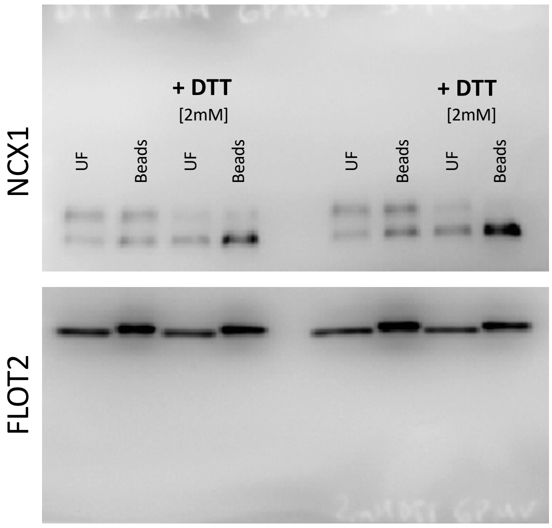

Supplementary Figure 1, Related to STAR Methods. The presence of DTT during GPMV preparation does not influence palmitoylation of NCX1 or flotillin 2. Palmitoylated proteins were prepared by resin-assisted capture of acylated proteins with or without pretreatment of lysates with 2mM DTT. The presence of DTT does not alter the quantity of NCX1 captured by the assay, but it does reduce intramolecular aggregation of NCX1 (the upper band on the western blot) during sample preparation. UF: unfractionated cell lysate, Beads: palmitoylated proteins.

Sequence of Peptides	
DHHC5 213-237	biotin-RGRTTNEQVTGKFRGGVNPFTNGCC
DHHC5 223-247	biotin-GKFRGGVNPFTNGCCNNVSRVLCSS
DHHC5 233-257	biotin-TNGCCNNVSRVLCSSPAPRYLGRPK
DHHC5 243-267	biotin-VLCSSPAPRYLGRPKKEKTIVIRPP
DHHC5 253-277	biotin-LGRPKKEKTIVIRPPFLRPEVSDGQ
DHHC5 263-287	biotin-VIRPPFLRPEVSDGQITVKIMDNGI
DHHC5 273-297	biotin-VSDGQITVKIMDNGIQGELRRTKSK
DHHC5 283-307	biotin-MDNGIQGELRRTKSKGSLEITESQS
DHHC5 293-317	biotin-RTKSKGSLEITESQSADAEPPPPPK
DHHC5 303-327	biotin-TESQSADAEPPPPPKPDLSRYTGLR
DHHC5 313-337	biotin-PPPPKPDLSRYTGLRTHLGLATNED
DHHC5 323-347	biotin-YTGLRTHLGLATNEDSSLLAKDSPP
DHHC5 333-357	biotin-ATNEDSSLLAKDSPPTPTMYKYRPG
NCX1 XIP with biotin	biotin-RRLLFYKYVYKRYRAGKQRG
NCX1 XIP without biotin	RRLLFYKYVYKRYRAGKQRG
NCX1 K229Q XIP	biotin-RRLLFYKYVYQRYRAGKQRG
NCX1 619-643	KYLFGQPVFRKVHAREHPILSTVIT
NCX1 629-653	KVHAREHPILSTVITIADEYDDKQP
NCX1 639-663	STVITIADEYDDKQPLTSKEEEERR
NCX1 649-673	DDKQPLTSKEEEERRIAEMGRPILG
NCX1 659-683	EEERRIAEMGRPILGEHTKLEVIIE
NCX1 669-693	RPILGEHTKLEVIIEESYEFKSTVD
NCX1 679-703	EVIIEESYEFKSTVDKLIKKTNLAL
NCX1 689-713	KSTVDKLIKKTNLALVVGTNSWREQ
NCX1 699-723	TNLALVVGTNSWREQFIEAITVSAG
NCX1 709-733	SWREQFIEAITVSAGEDDDDDECGE
NCX1 719-743	TVSAGEDDDDDECGEEKLPSCFDYV
NCX1 729-753	DECGEEKLPSCFDYVMHFLTVFWKV
NCX1 739-763	CFDYVMHFLTVFWKVLFAFVPPTEY
NCX1 742-766	YVMHFLTVFWKVLFAFVPPTEYWNG
NCX1 740-756	biotin-FDYVMHFLTVFWKVLFA

Supplemental Table 2, Related to STAR Methods. Sequences of peptides used in this investigation
