# Supplementary material for: Dynamic Palmitoylation of the Sodium-Calcium Exchanger Modulates Its Structure, Affinity for Lipid-Ordered Domains, and Inhibition by XIP
Source: Cell Rep. 2020 Jun 9;31(10):107697. doi: 10.1016/j.celrep.2020.107697 (PMC7296346; doi:10.1016/j.celrep.2020.107697)
Supplement: Document S2. Article plus Supplemental Information [file mmc3.pdf]

Cell Reports

Article

Dynamic Palmitoylation of the Sodium-Calcium Exchanger Modulates Its Structure, Affinity for Lipid-Ordered Domains, and Inhibition by XIP

Graphical Abstract

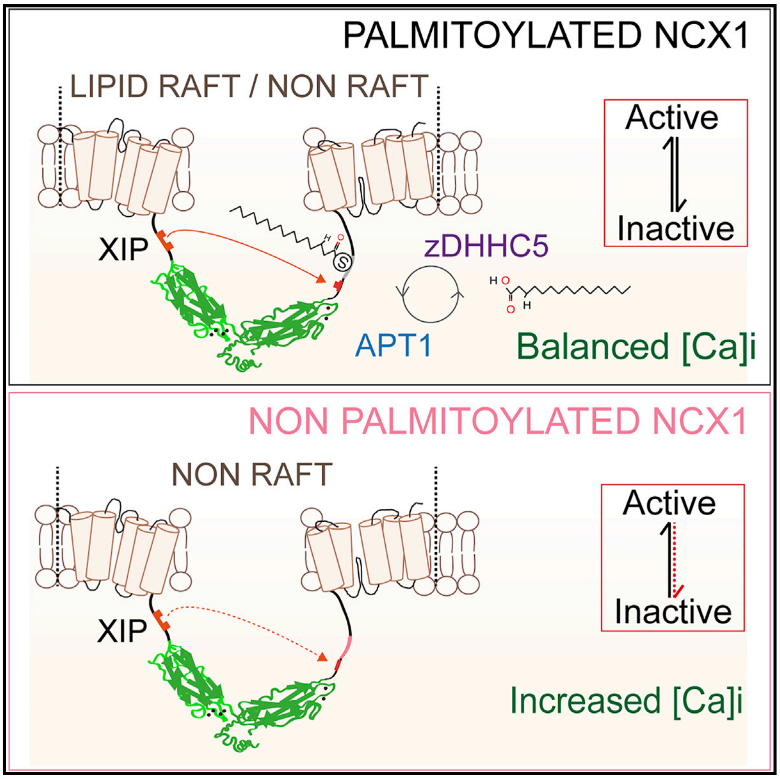

Authors

Caglar Gök, Fiona Plain, Alan D. Robertson, Jacqueline Howie, George S. Baillie, Niall J. Fraser, William Fuller

Correspondence

will.fuller@glasgow.ac.uk

In Brief

Gök et al. comprehensively define the cellular control and molecular consequences of NCX1 palmitoylation. They identify the binding site for the endogenous XIP domain in the NCX1 regulatory loop and demonstrate that, by changing XIP binding to this site, palmitoylation controls NCX1-mediated transmembrane Ca flux and hence cytosolic Ca levels.

Highlights

- NCX1 is dynamically palmitoylated at the cell surface by zDHHC5 and APT1
- Palmitoylation modifies the NCX1 dimer's structure and affinity for lipid rafts
- We identify the binding site of the endogenous XIP domain in NCX1's regulatory loop
- Palmitoylation modifies NCX1 XIP affinity and hence regulates intracellular Ca

Gök et al., 2020, Cell Reports *31*, 107697
June 9, 2020 © 2020 The Authors.
https://doi.org/10.1016/j.celrep.2020.107697

CellPress

Cell Reports

CellPress
OPEN ACCESS

Article

Dynamic Palmitoylation of the Sodium-Calcium Exchanger Modulates Its Structure, Affinity for Lipid-Ordered Domains, and Inhibition by XIP

Caglar Gök,[1] Fiona Plain,[2] Alan D. Robertson,[1] Jacqueline Howie,[1] George S. Baillie,[1] Niall J. Fraser,[2] and William Fuller[1,3,4,*]
[1]Institute of Cardiovascular & Medical Sciences, Sir James Black Building, University of Glasgow, Glasgow G12 8QQ, UK
[2]School of Medicine, Ninewells Hospital, University of Dundee, Dundee DD1 9SY, UK
[3]Lead Contact
[4]Twitter: @FullerLabGlas
*Correspondence: will.fuller@glasgow.ac.uk
https://doi.org/10.1016/j.celrep.2020.107697

SUMMARY

The transmembrane sodium-calcium (Na-Ca) exchanger 1 (NCX1) regulates cytoplasmic Ca levels by facilitating electrogenic exchange of Ca for Na. Palmitoylation, the only reversible post-translational modification known to modulate NCX1 activity, controls NCX1 inactivation. Here, we show that palmitoylation of NCX1 modifies the structural arrangement of the NCX1 dimer and controls its affinity for lipid-ordered membrane domains. NCX1 palmitoylation occurs dynamically at the cell surface under the control of the enzymes zDHHC5 and APT1. We identify the position of the endogenous exchange inhibitory peptide (XIP) binding site within the NCX1 regulatory intracellular loop and demonstrate that palmitoylation controls the ability of XIP to bind this site. We also show that changes in NCX1 palmitoylation change cytosolic Ca. Our results thus demonstrate the broad molecular consequences of NCX1 palmitoylation and highlight a means to manipulate the inactivation of this ubiquitous ion transporter that could ameliorate pathologies linked to Ca overload via NCX1.

INTRODUCTION

The sodium-calcium (Na-Ca) exchanger 1 (NCX1) regulates intracellular Na and Ca by facilitating the bidirectional transport of Ca under the control of the transmembrane Na gradient in excitable and non-excitable cells. In cardiac muscle, for example, NCX1 activity is an important determinant of ventricular filling, but it also indirectly controls systolic function by competing with the enzyme responsible for intracellular Ca storage, SERCA2a (Ottolia et al., 2013). Inappropriate NCX1 activity contributes to numerous cardiac pathologies, including myocardial infarction (Ohtsuka et al., 2004), heart failure (Flesch et al., 1996), and arrhythmias (Dai et al., 2019; Voigt et al., 2012).

NCX1 is a functional dimer (John et al., 2011; Ren et al., 2008) that is allosterically regulated by both ions it transports through effects on its large regulatory intracellular loop (f-loop). Elevated intracellular Na inactivates NCX1 (Hilgemann et al., 1992), but the identity of the regulatory Na binding site responsible for this inactivation is not known. The binding of Ca at two Ca-binding domains (CBDs) counteracts this inhibition and activates NCX1 (Ottolia et al., 2009). Binding of 4 Ca ions to CBD1, the high-affinity Ca -binding site (K_d ~0.2 μM), activates NCX1; binding of 2 Ca ions to CBD2, a lower-affinity site (K_d ~10 μM), opposes NCX1 inactivation (Khananshvili, 2020). Structural information to explain how allosteric regulation via occupancy of the CBDs is translated into functional effects on ion transport remains poorly understood, as the only exchanger to have its structure solved lacks allosteric regulatory sites (Liao et al., 2012, 2016).

The activities of numerous ion channels and transporters are key to controlling the function of excitable tissues. Voltage-gated channels, ion pumps, exchangers, and their accessory subunits are all palmitoylated (Howie et al., 2013; Shipston, 2011), with diverse functional consequences. Palmitoylation is the only reversible post-translational modification reported to regulate NCX1 (Fuller et al., 2016; Gök and Fuller, 2020; Reilly et al., 2015). S-palmitoylation, the esterification of palmitate to cysteine side chains, reversibly anchors intracellular regions of proteins to membranes (Linder and Deschenes, 2007). Catalyzed by zinc-finger- and DHHC-motif-containing palmitoyl acyl transferases (zDHHC-PATs; Mitchell et al., 2006), and reversed by thioesterases, this lipid modification regulates the activity, stability, subcellular distribution, and lipid interactions of peripheral and integral membrane proteins (Salaun et al., 2010). Palmitoylation can control the affinity of proteins for membranes based on their shape (Larsen et al., 2015); it directly influences membrane curvature (Chlanda et al., 2017) and promotes the clustering and internalization of both proteins and lipids (Hilgemann et al., 2013). It is therefore important to understand the control and cellular consequences of this post-translational modification for protein structure and function.

This is an open access article under the CC BY license (http://creativecommons.org/licenses/by/4.0/).

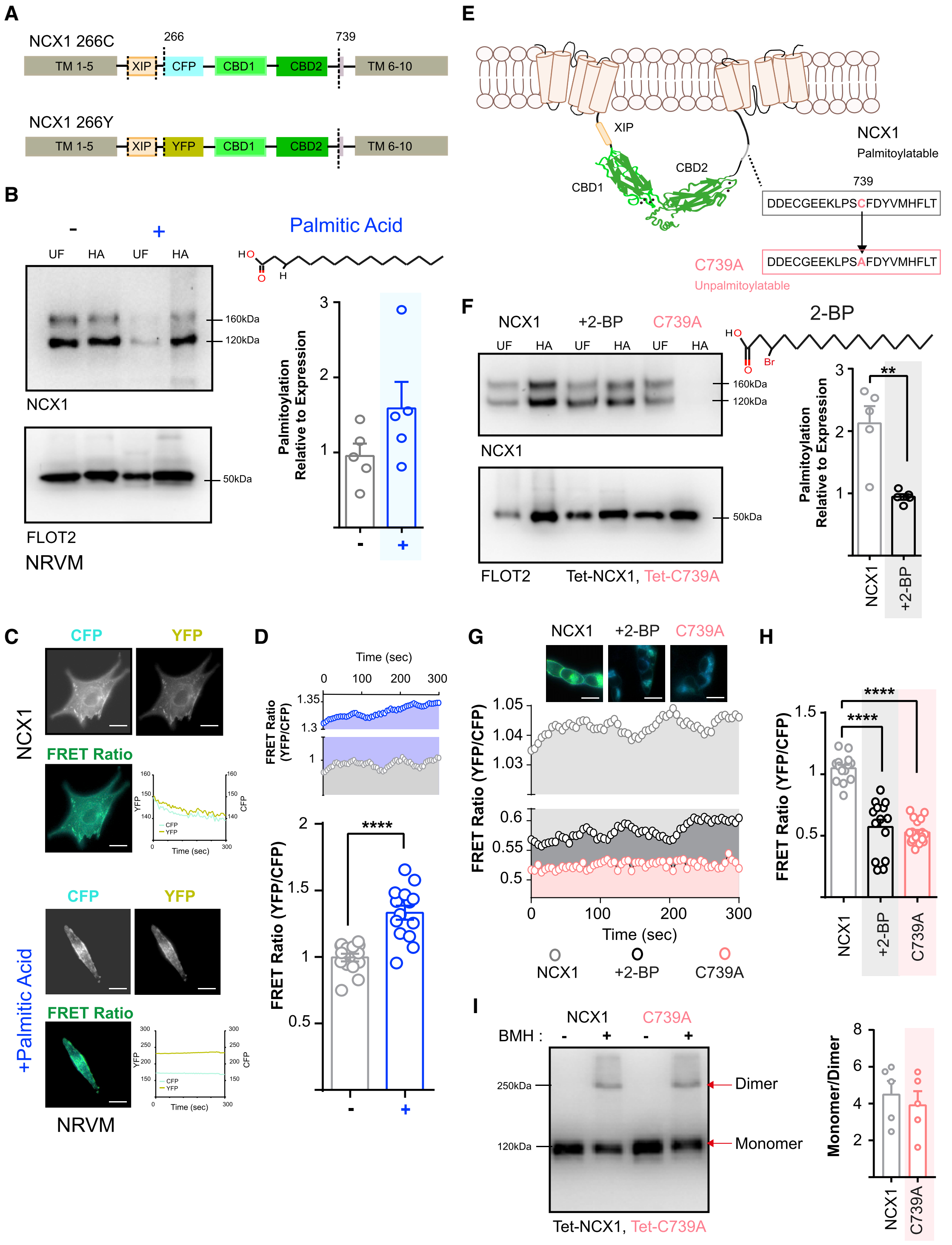

Figure 1. Palmitoylation Modifies FRET between NCX1 Dimers

(A) Schematic of the NCX1 FRET sensors used in this investigation, indicating the positions of transmembrane (TM) domains, exchange inhibitory peptide (XIP), FRET sensors (CFP and YFP), Ca binding domains (CBDs), and palmitoylation site.

(B) Palmitic acid (upper structure, at 20 μM, 4 h) supplementation increases the palmitoylation of endogenous NCX1 in neonatal rat ventricular myocytes (NRVMs). Western blots show abundance of NCX1 (upper) and the lipid raft resident protein flotillin 2 (loading control, lower) in unfractionated cell lysates (UF) and purified palmitoylated fraction (HA). The bar chart (right) shows NCX1 palmitoylation (HA fraction) normalized to expression (UF) in treated (blue, +) relative to untreated (–) NRVMs (N = 5).

(legend continued on next page)

NCX1 is palmitoylated at a single cysteine at position 739 in its f-loop, on the carboxyl terminal side of CBD2 (Fuller et al., 2016; Gök and Fuller, 2020; Plain et al., 2017; Reilly et al., 2015). Palmitoylation is required for NCX1 inactivation, which is mediated by the interaction of the endogenous exchange inhibitory peptide (XIP) with the f-loop. XIP inactivation of NCX1 requires residues 562–679 (Maack et al., 2005; Matsuoka et al., 1993), although this large region of the NCX1 f-loop includes part of CBD2 and it is likely that the core XIP-binding site is smaller. The physiological importance of NCX1 inactivation has not yet been fully established. Inactivation likely represents a means to tune NCX1 activity in response to mobilization of PIP2 (which usually sequesters the XIP domain). Furthermore, NCX1 inactivation plays an important role in limiting NCX1-mediated Ca influx (and hence cellular injury) under circumstances of Na overload, metabolic stress, or acidosis (Hilgemann et al., 2006; Reeves and Condrescu, 2008).

Molecular recognition of the NCX1 palmitoylation site is facilitated by the presence of an amphipathic α-helix, which is positioned at the carboxyl terminal end of the f-loop (Plain et al., 2017). However, how palmitoylation regulates NCX1 behavior is only partially understood. Furthermore, it remains unknown whether NCX1 palmitoylation is a one-off event, linked to its passage through the secretory pathway, or a dynamic event that occurs at the cell surface. Here, we investigated how palmitoylation regulates the dimerization, lipid interactions, and inactivation of NCX1 in HEK cells and ventricular myocytes and report several important advances. Our findings show that palmitoylation modifies both NCX1-NCX1 dimerization and the ability of XIP to bind to and inactivate NCX1. We identify a region of the NCX1 f-loop adjacent to the NCX1 palmitoylation site where XIP binds, which is distinct from the region of the f-loop required for XIP to inactivate NCX1. We find that palmitoylation of NCX1 at the cell surface is dynamic and that the structural motif in the NCX1 f-loop that is required for NCX1 palmitoylation also recruits the thioesterase APT1. From our findings, we propose that palmitoylation creates a site within the NCX1 f-loop where XIP binds, thereby facilitating NCX1 inactivation while also controlling the protein's affinity for lipid rafts in the plasma membrane. The reduced sensitivity of non-palmitoylated NCX1 to inactivation ultimately leads to elevated intracellular Ca. Hence, the palmitoylation status of NCX1 regulates cytosolic Ca.

RESULTS

Palmitoylation Increases NCX1-NCX1 FRET Signals in Live Cells

Previously, NCX1 dimerization has been detected by performing intermolecular fluorescence resonance energy transfer (FRET) between CFP- and YFP-tagged NCX1 in plasma membrane sheets prepared from *Xenopus* oocytes (John et al., 2011). Here, we expressed full-length NCX1 with the same fluorophores inserted at position 266 (at the N-terminal end of the NCX1 f-loop; Figure 1A) in neonatal rat ventricular myocytes (NRVMs). Palmitic acid supplementation of myocytes is known to enhance the palmitoylation of certain cardiac proteins (Pei et al., 2016). The treatment of NRVMs with palmitic acid increased both endogenous NCX1 palmitoylation (Figure 1B) and NCX1-NCX1 FRET (Figures 1C and 1D). These palmitoylation-dependent changes in NCX1 FRET behavior suggest that either (1) palmitoylation regulates NCX1 dimerization or (2) palmitoylation restructures the f-loop in existing NCX1 dimers to promote intermolecular FRET.

Next, we evaluated NCX1 FRET activity in HEK cells that expressed either wild-type (WT) or unpalmitoylatable (C739A) NCX1 (Figures 1E and 1F). The FRET signal generated by unpalmitoylatable NCX1 was markedly reduced compared to that of WT NCX1 (Figure 1G). Furthermore, the broad-spectrum zDHHC-PAT inhibitor 2-bromopalmitate significantly reduced both NCX1 palmitoylation and FRET between WT NCX1 dimers (Figures 1G and 1H). In both cell types tested, NCX1 palmitoylation consistently caused an increase in NCX1-NCX1 FRET signals. The intensity of any FRET signal depends considerably on the distance ($1/r^6$) between donor and acceptor fluorophores. The observed increase in NCX1-NCX1 FRET that occurs following NCX1's palmitoylation most likely reflects a reduction in the distance between the two fluorophores. This, in turn, suggests that the palmitoylation of the f-loop induces a discernible conformational change within the cytoplasm-facing structure of NCX1. To determine whether the change in NCX1-NCX1 FRET signal induced by palmitoylation reflected a conformational change in existing dimers or a difference in the propensity of NCX1 to dimerize, FT-293 cells expressing either WT or unpalmitoylatable NCX1 were briefly treated with the homo-bifunctional cross-linker bismaleimidohexane (BMH). The amount of NCX1 dimer detected was essentially the same (Figure 1I). We conclude that palmitoylation restructures existing NCX1 dimers.

(C) NCX1-NCX1 FRET measurements in transiently transfected NRVMs. The images show representative cells visualized in the CFP and YFP channels. The FRET ratio was calculated as the ratio of background-subtracted −YFP and −CFP signals (scale bar, 10 μm).

(D) Palmitic acid supplementation (20 μM, 4 h) significantly enhances NCX1-NCX1 FRET in treated (+) relative to untreated (−) NRVMs. ****$p < 0.0001$, calculated by unpaired t test. N = 14.

(E) Position of the NCX1 palmitoylation site. The magnified box shows the position of the C739A mutation, which prevents the palmitoylation of NCX1.

(F) FT-293 cells that stably express tetracycline (Tet)-inducible WT NCX1 treated with 2-bromopalmitate (2-BP; 50 μM, 4 h) showed reduced NCX1 palmitoylation. In FT-293 cells that stably express Tet-inducible C739A NCX1, NCX1 is not palmitoylated. The bar chart (right) shows NCX1 palmitoylation normalized to expression, in 2-BP-treated (black) relative to untreated (gray) FT-293 cells. **$p = 0.003$, calculated by unpaired t test. N = 5.

(G) An example of NCX1-NCX1 FRET measurements in transiently transfected HEK293 cells expressing WT NCX1 (left), WT NCX1 in the presence of 2-BP (50 μM, 4 h, middle), and C739A NCX1 (right). Scale bar, 10 μm.

(H) NCX1-NCX1 FRET activity is significantly reduced in HEK293 cells expressing WT NCX1 and treated with 2-BP (50 μM, 4 h) and in HEK293 cells expressing C739A NCX1. ****$p < 0.0001$, calculated by unpaired t test. N = 14 (WT), 14 (WT+2-BP), and 19 (C739A).

(I) Cross-linking of NCX1 using 0.1 mM BMH. The NCX1 monomer migrates at ~120 kDa and the dimer at ~250 kDa. The monomer/dimer ratio was identical between palmitoylatable WT NCX1 and unpalmitoylatable C739A. N = 5 for WT NCX1 and C739A.

Lipid Interactions of Palmitoylated NCX1

The affinity of single-transmembrane-domain proteins for lipid rafts is regulated by palmitoylation (Lorent et al., 2017), and numerous integral membrane proteins are palmitoylated and reside in lipid rafts (Wypijewski et al., 2015). However, to date, no multi-pass membrane domain protein has been found to have a significant affinity for the raft phase formed in phase-separated giant plasma membrane vesicles (GPMVs), which are widely used to investigate membrane protein behaviors in lipid raft and non-raft phases (Diaz-Rohrer et al., 2014; Levental et al., 2010; Lorent et al., 2017; Sezgin et al., 2012). Here, we investigated the effect of palmitoylation on the affinity of NCX1 for lipid rafts in intact cells and GPMVs. We treated HEK cells with 5 mM methyl-β-cyclodextrin (MβCD) to disrupt membrane rafts and with 25 μM SDS to enhance raft formation. MβCD removes cholesterol from biological membranes, while low concentrations of detergents, such as SDS, enhance raft formation in intact cells by increasing disorder in disordered microdomains (Hilgemann et al., 2018; Zhou et al., 2013). Cells were stained with filipin to confirm the successful depletion or clustering of cholesterol following MβCD or SDS treatment (Figure 2A). Enhancing raft formation with SDS significantly increased NCX1-NCX1 FRET signals, but only when NCX1 was palmitoylatable (Figure 2B). The disruption of lipid rafts did not alter the FRET signals produced by unpalmitoylatable NCX1, but it did reduce FRET signals between WT NCX1 dimers such that they were indistinguishable from that of unpalmitoylated NCX1 (Figure 2C). That the higher steady-state FRET between WT NCX1 dimers is sensitive to the destruction of lipid rafts suggests that palmitoylation enhances the affinity of NCX1 for rafts. We explored this possibility by visualizing the distribution of CFP-tagged NCX1 in phase-separated GPMVs (Figure 2D). Unpalmitoylatable NCX1 partitioned entirely to the non-raft phase upon phase separation. Interestingly, the presence of palmitoylated WT NCX1 impaired the phase separation of GPMVs, which suggests that palmitoylation of NCX1 fundamentally alters the behavior of the membrane. WT NCX1 colocalized with both raft and non-raft markers in GPMVs (Figure 2E), indicating that palmitoylation does indeed control the affinity of NCX1 for lipid rafts.

NCX1 Palmitoylation Creates an XIP Binding Site within the f-Loop

NCX1 is inactivated when XIP (residues 219–238), located at the N-terminal end of the NCX1 f-loop, interacts with a distal region of the same loop (residues 562–679; Maack et al., 2005; Figure 3A). Palmitoylation enhances NCX1's inactivation, but the molecular basis of this process is only partially understood. We investigated whether palmitoylation modifies the relationship between XIP and NCX1, using a WT or mutant XIP peptide. The point mutant XIP peptide we used cannot inactivate NCX1 (K229Q; Matsuoka et al., 1997). We also depleted PIP2 pharmacologically (Figure 3B), as PIP2 usually sequesters the endogenous XIP domain to prevent NCX1 inactivation. We found that biotinylated WT and K229Q XIP peptides could affinity purify WT NCX1 from cell lysates with equal efficiency (Figure 3C), which suggests that XIP engagement with and inactivation of NCX1 are separate events. By fusing YFP to regions of the NCX1 f-loop (Plain et al., 2017), we probed the XIP binding site in the f-loop (Figure 3D). Biotinylated XIP affinity purified NCX1 370–765, 501–765, 599–765, and surprisingly 690–765, which does not include any part of the f-loop necessary for XIP to inactivate NCX1 (Maack et al., 2005). Again, this emphasizes the separation of binding and efficacy in XIP inactivation of NCX1.

Next, we investigated the role of palmitoylation in regulating XIP interaction with the NCX1 f-loop. Biotinylated XIP peptide showed a substantially reduced ability to pull down unpalmitoylatable NCX1 following affinity purification (Figure 3E). This suggests that NCX1 undergoes a conformational change on being palmitoylated, which creates a binding site within the f-loop for XIP. We sought to identify the location of this binding site by probing an array of overlapping 25-mer peptides representing NCX1 619–766 (covering the C-terminal half of CBD2 and the linker between CBD2 and TMD6) with biotinylated XIP. We took advantage of the fact that 15 μM, but not 5 μM, XIP binds to the f-loop (Figure 3E) and classified peptides in the array interacting with 5 μM XIP as false positives. Both 5 μM and 15 μM XIP interacted with peptides representing the C-terminal end of CBD2 (659–713, including the final Ca-coordinating residue, E684). 15 μM XIP uniquely interacted with 25-mer peptides covering 699–743, suggesting that the core XIP binding site is a region immediately on the N-terminal side of the palmitoylation site, 709-SWREQFIEAITVSAGEDDDD-728 (Figure 3F). We therefore deleted this region from the NCX1 f-loop and tested the impact on XIP binding. Since NCX1 palmitoylation effects XIP binding, we first confirmed that palmitoylation of YFP-NCX1 266–765 was unaffected by the mutation Δ709–728 (Figure 3G). Affinity purification of YFP-NCX1 266–765 by biotinylated XIP peptide was largely abolished by the mutation Δ709–728 (Figure 3H). Hence, we conclude that XIP interacts with NCX1 709–728.

Since XIP is rich in basic amino acids, we reasoned that it would cross the cell membrane in the same manner as reported for other cell-penetrant polybasic peptides (Milletti, 2012). We therefore investigated the impact on NCX1 FRET activity of applying XIP to intact cells. WT, but not K229Q, XIP reduced the WT NCX1 FRET signal; the FRET activity of unpalmitoylatable NCX1 was modestly increased by XIP (Figure 3I). We depleted PIP2 by activating phospholipase C with 3M3FBS in the presence of the phosphatidylinositol 3-kinase (PI3K) inhibitor wortmannin to prevent PIP2 resynthesis and then measured NCX FRET. This pharmacological release of XIP from PIP2 also reduced the FRET activity of WT NCX1 (Figure 3I). We concluded from these results that the binding of XIP to NCX1 and NCX1 inactivation are independent events. XIP can bind to NCX1 regardless of whether or not it is capable of inactivating it (XIP K229Q), but NCX1 needs to be palmitoylated to interact with XIP with a high affinity.

NCX1 Depalmitoylation Pathways

Palmitoylation of NCX1 at C739 requires an amphipathic α-helix (residues 740–756) located adjacent to this cysteine (Plain et al., 2017). We reasoned that the zDHHC-PAT responsible for palmitoylating NCX1 might interact with this helix. We therefore used a peptide that represents NCX1 amino acids 740–756 and carries an N-terminal biotin tag to affinity purify interacting proteins from

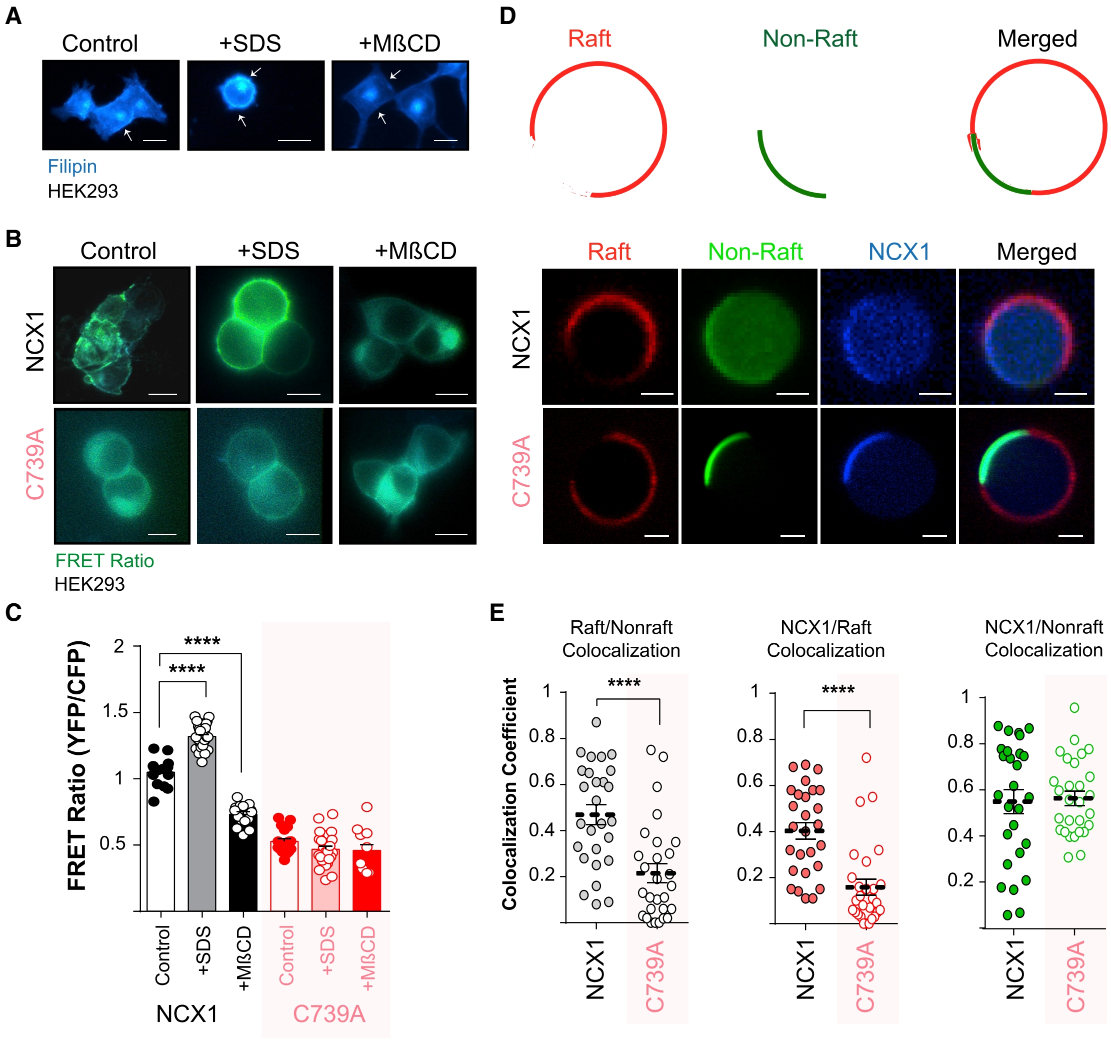

Figure 2. NCX1 Localization to Lipid Microdomains

(A) Visualization of cellular cholesterol with filipin in control (left), SDS-treated (center), and MβCD-treated (right) HEK293 cells (scale bar, 20 μm).

(B) Representative FRET images in HEK293 cells expressing WT (upper) and unpalmitoylatable (C739A, lower) NCX1 FRET sensors following treatment to enhance (SDS) or reduce (MβCD) raft formation (scale bar, 10 μm).

(C) Mean FRET data. Enhancing raft formation increases NCX1-NCX1 FRET and reducing raft formation decreases NCX1-NCX1 FRET, but only when NCX1 is palmitoylatable. ****$p < 0.0001$, calculated by unpaired t test. N = 14 (WT), 35 (WT+SDS), 16 (WT+MβCD), 19 (C739A), 26 (C739A+SDS), and 14 (C739A+MβCD).

(D) Schematic representation of giant plasma membrane vesicle (GPMV) phase separation and representative images displaying phase-separation behavior in the presence of WT NCX1 (upper) and C739A NCX1 (lower). Scale bar, 2 μm.

(E) Colocalization analysis in phase-separated GPMVs. Left: increased colocalization of raft (CTxB-Alexa Fluor 647) and non-raft (FAST-DIL) markers in the presence of WT versus C739A NCX1. Center: raft colocalization of WT and unpalmitoylatable NCX1. Right: non-raft colocalizaton of WT and unpalmitoylatable NCX1. ****$p < 0.0001$, calculated by unpaired t test. N = 27 (WT) and 28 (C739A).

rat cardiac and brain lysates (Figure 4A). The interacting proteins were then identified by liquid chromatography-tandem mass spectrometry (LC-MS/MS) (Table S1). No zDHHC-PATs were found to interact with this peptide. However, we did identify the depalmitoylating enzyme APT1 (LYPLA1) from heart lysates. We therefore investigated whether APT1 depalmitoylates NCX1. The broad-spectrum thioesterase inhibitor palmostatin B (PalmB; which inhibits ABHD isoforms as well as APT1; Lin and Conibear, 2015) significantly enhanced both NCX1 palmitoylation and FRET between WT NCX1 dimers in HEK cells (Figures 4B and 4C). The specific APT1 inhibitor ML348 (Adibekian et al., 2012) also enhanced NCX1 palmitoylation and FRET, but the APT2 inhibitor ML349 did not. PalmB and ML348, but not ML349, also increased NCX1 palmitoylation in adult rabbit ventricular myocytes (Figure 4B). None of these drugs significantly influenced FRET between unpalmitoylatable NCX1 dimers (not shown). These experiments show that the palmitoylation of NCX1 is dynamic, with depalmitoylation depending on the activity of APT1, but not APT2. Our results therefore suggest that APT1 is likely to be the enzyme that mediates NCX1

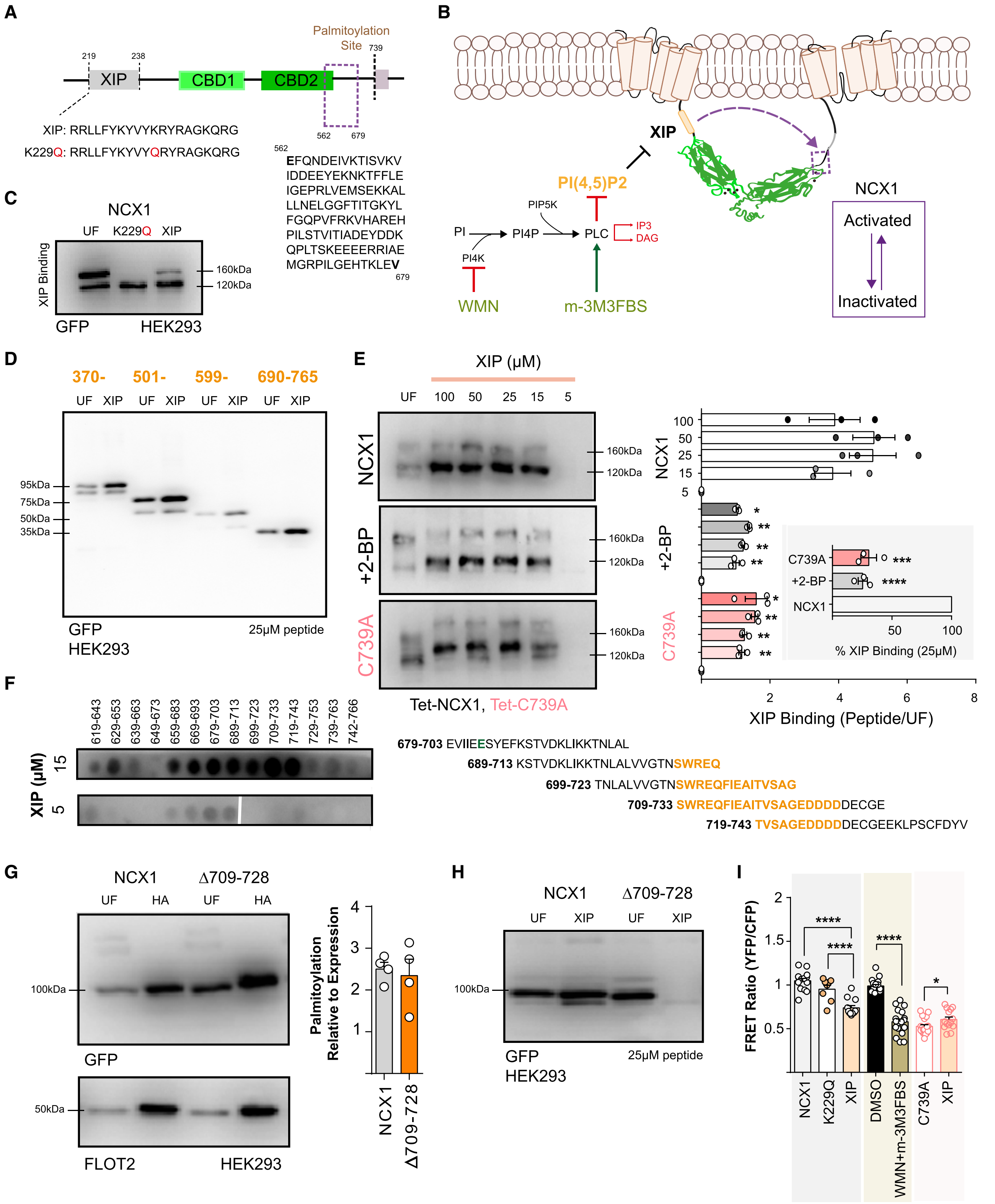

Figure 3. Palmitoylation Enhances NCX1 XIP Affinity

(A) Schematic of the NCX1 intracellular f-loop, highlighting the position of XIP, CBDs, and the region of the f-loop required for XIP inactivation of NCX1 (562–679) near the palmitoylation site.

(legend continued on next page)

depalmitoylation. Moreover, the region of NCX1 required for its palmitoylation may also be responsible for recruiting the NCX1 thioesterase.

NCX1 Palmitoylation Status and Cellular Environment

We investigated whether allosteric regulation by Na or Ca influenced NCX1-NCX1 FRET by removing these ions from the extracellular solution (Figure 5A). In Na-free solutions, NCX1-NCX1 FRET was substantially reduced, and in Ca-free solutions, NCX1-NCX1 FRET was significantly increased (Figures 5B and 5C). Remarkably, these changes in FRET were not observed when NCX1 was unpalmitoylatable, which suggests that they are mediated by changes in NCX1 palmitoylation (Figures 5B and 5C). Indeed, we found that Na-free solutions reduced and Ca-free solutions enhanced NCX1 palmitoylation (Figure 5D). These experiments provide additional support for NCX1 being dynamically palmitoylated following its delivery to the cell surface. They furthermore suggest that the NCX1 Ca-free (Na-bound) structure is preferentially palmitoylated and the Na-free (Ca-bound) structure is preferentially depalmitoylated.

zDHHC5 Palmitoylates NCX1 at the Cell Surface

The cell-surface zDHHC5-PAT recruits substrates via its extended intracellular C tail (Howie et al., 2014). We used a library of overlapping biotinylated peptides, which represent the first section of this C tail, to identify zDHHC5-binding proteins in rat cardiac lysates and found that NCX1 interacts with zDHHC5 (Figure 6A). To investigate a role for zDHHC5 in the palmitoylation of NCX1, we generated zDHHC5 knockout (KO) cells by transfecting FT-293 cells that express tetracycline-inducible cas9 with a guide RNA targeting zDHHC5 (Munoz et al., 2014). Successful gene targeting was confirmed by sequencing a region of PCR-amplified zDHHC5 exon 2 (bases 57,672,929 to 57,673,619 of *Homo sapiens* chromosome 11; the zDHHC5 initiator methionine is the ATG in position 57,673,091). zDHHC5 protein was undetectable in lysates from KO cells (data not shown).

We first confirmed that zDHHC5 palmitoylates NCX1 by co-expressing WT zDHHC5 or catalytically inactive zDHHS5 with NCX1 in zDHHC5 KO cells (Figure 6B). FRET between WT NCX1 dimers was substantially reduced in zDHHC5 KO compared to WT HEK cells (Figures 6C and 6D). When zDHHC5 KO cells were exposed to Ca-free extracellular solutions, there was no change in FRET between WT NCX1 dimers (Figures 6C and 6D). This is therefore consistent with the notion that zDHHC5 is the "sensor" that mediates dynamic changes in NCX1 palmitoylation and FRET in different cellular environments.

NCX1 Palmitoylation and the Control of Intracellular Ca

To date, the impact of palmitoylation on NCX1 activity has been defined using whole-cell voltage clamp following the dialysis of intracellular contents. Given our finding that palmitoylation changes XIP affinity of NCX1 and occurs dynamically at the cell surface, we evaluated the impact of NCX1 palmitoylation on intracellular Ca in intact FT-293 cells loaded with the Ca-sensitive dye Fluo 4. Under physiological conditions (150 mM Na, 1.8 mM extracellular Ca, 10 mM Na, 100 nM intracellular Ca), the reversal potential of NCX1 is 42 mV. In excitable tissues (resting membrane potential, −80 mV), NCX1-mediated Na influx and Ca efflux occur at rest, but in HEK cells (resting membrane potential, −24 mV; Kirkton and Bursac, 2011), NCX1 acts as a Ca-influx pathway. Intracellular Ca was higher in the presence of unpalmitoylatable NCX1 compared to WT NCX1 (Figure 7A). This implies greater NCX-mediated Ca influx occurs when NCX1 is not palmitoylated as a result of its reduced XIP sensitivity. In separate experiments, increasing NCX1 palmitoylation with PalmB reduced intracellular Ca in FT-293 cells expressing WT NCX1 (Figure 7B), again suggesting that the palmitoylation status of NCX1 controls intracellular Ca.

DISCUSSION

In this paper, we set out to investigate the cellular mechanisms controlling NCX1 palmitoylation and the consequences of palmitoylation for NCX1 dimerization and lipid interactions, as well as to understand the molecular basis of the requirement for palmitoylation for NCX1 to inactivate. Our findings show that the relationship between NCX1 and the lipid bilayer in which it resides is fundamentally altered by palmitoylation. Our results show, for the first time, that the palmitoylation of NCX1 at the cell surface occurs dynamically under the control of APT1 and zDHHC5, with the rates of palmitoylation and depalmitoylation influenced by the NCX1 conformational poise. Palmitoylation increases FRET

(B) Schematic of the relationship between XIP and PIP2, highlighting the drugs (WMN and m-3M3FBS) used to manipulate PIP2 concentrations.

(C) Biotinylated WT and inactive (K229Q) XIP peptides affinity purify YFP-tagged WT NCX1 f-loop equally efficiently from HEK cell lysates.

(D) Biotinylated WT XIP peptides affinity purify YFP-tagged NCX1 f-loop regions 370–765, 501–765, 599–765, and 690–765 from HEK cell lysates. UF, unfractionated cell lysate; XIP, proteins affinity purified by biotinylated XIP.

(E) Affinity purification of NCX1 from FT-293 cells expressing full-length NCX1 by a biotinylated XIP peptide is substantially reduced when NCX1 palmitoylation is reduced (2-BP) or abolished (C739A). Bar charts (right) show the amount of NCX1 that co-purified with 5–100 μM XIP, relative to the abundance of NCX1 in the unfractionated cell lysate (UF). Statistical comparisons are between the relative amounts of WT, 2-BP-treated, and C739A NCX1 co-purified by a particular concentration of XIP (p values range from 0.04 [*] to 0.008 [**], N = 3). Inset: comparison of the amount of NCX1 binding to 25 μM XIP from WT (defined as 100%), 2-BP-treated WT, or C739A NCX1-expressing cells. ****$p < 0.0001$, ***$p = 0.0005$ calculated by unpaired t test.

(F) Peptide array to map the XIP binding site in the NCX1 f-loop. Arrays of 25-mer peptides synthesized on membranes were probed with the indicated concentration of biotinylated XIP. The alignment alongside the arrays highlights the final Ca coordinating residue of CBD2 (E684) in green and the core XIP binding site (709-SWREQFIEAITVSAGEDDDD-728) in orange.

(G) Deletion of the putative XIP binding site (Δ709–728) has no impact on NCX1 palmitoylation (N = 4).

(H) Deletion of residues 709–728 abolishes the interaction between the NCX1 f-loop and XIP.

(I) FRET activity of WT NCX1 is reduced by application of WT, but not K229Q XIP, or following depletion of PIP2. XIP modestly increases the FRET behavior of unpalmitoylatable NCX1. ****$p < 0.0001$, ***$p = 0.0006$, *$p = 0.034$, calculated by unpaired t test. N = 14 (WT), 8 (WT+K229Q), 12 (WT+XIP), 18 (DMSO), 18 (WT+WMN+3-M3FBS 19 (C739A), and 16 (C739A+XIP).

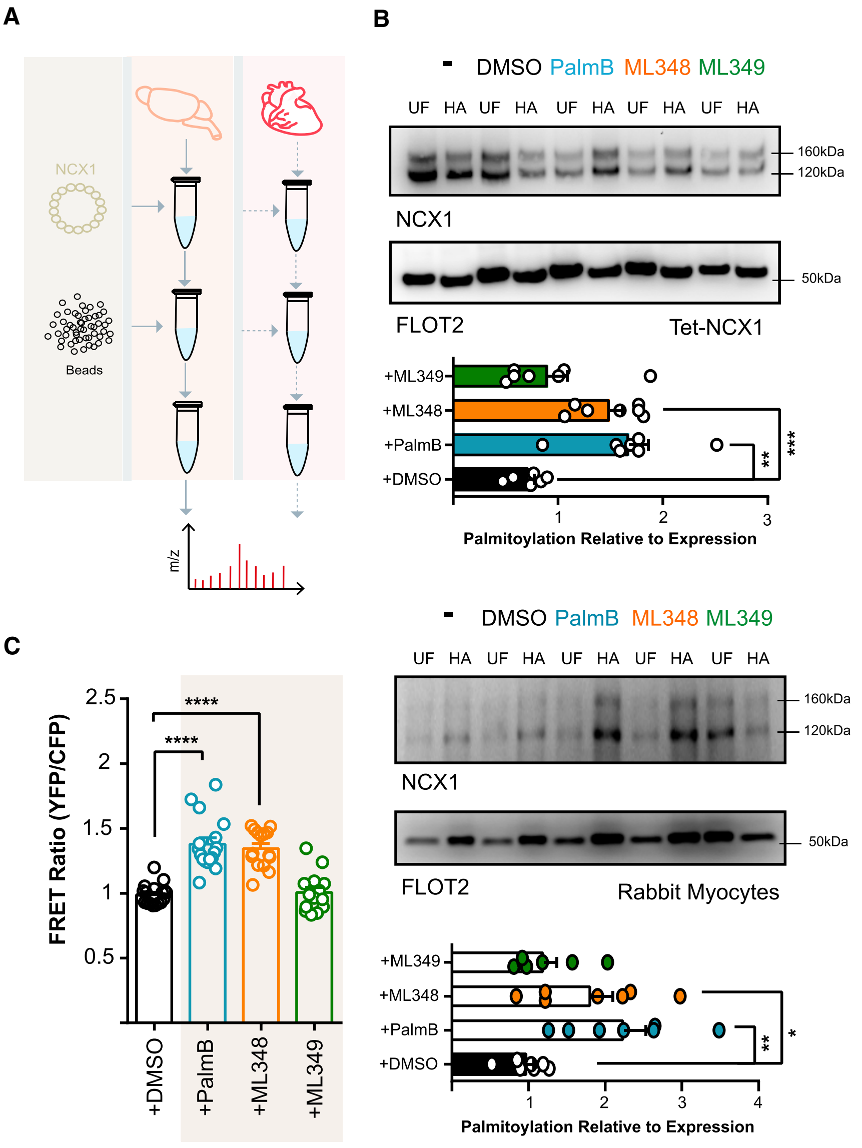

Figure 4. Identification of the NCX1 Depalmitoylating Enzyme

(A) Schematic of the affinity-purification reactions that identified APT1 as a possible NCX1-interacting protein.

(B) Impact of palmostatin B (PalmB; 20 μM), ML348 (APT1 inhibitor, 10 μM), and ML349 (APT2 inhibitor, 10 μM) on NCX1 palmitoylation in FT-293 cells stably expressing NCX1 (upper) and adult rabbit ventricular myocytes (lower). All drugs were applied for 3 h before measurements. The bar charts show NCX1 palmitoylation (HA fraction) normalized to expression (UF) following the indicated treatments. ***p = 0.0003, **p = 0.0015 (FT-293) and 0.0036 (rabbit myocytes), *p = 0.0261, calculated by unpaired t test. N = 7 (FT-293 cells), N = 7 (rabbit myocytes).

(C) Impact of the same thioesterase inhibitors on NCX1-NCX1 FRET. ****p < 0.0001, calculated by unpaired t test. N = 14 (untreated), 18 (DMSO) 17 (PalmB), 15 (ML348), and 14 (ML349).

between protomers within an NCX1 dimer, enhances the affinity of NCX1 for lipid-ordered domains at the cell surface, and is required for XIP to bind to and inactivate NCX1. Ultimately, by controlling the ability of NCX1 to inactivate, NCX1 palmitoylation regulates cytosolic Ca.

XIP, Inactivation, and Control of Intracellular Ca by NCX1 Palmitoylation

The functional effect of NCX1 palmitoylation is to modify NCX1 inactivation (Reilly et al., 2015). We have identified a region of the f-loop adjacent to the palmitoylation site (residues 709–728, enriched in negatively charged amino acids) that is required for XIP binding to the f-loop. This is consistent with an electrostatic interaction between the polybasic XIP sequence and this region of the f-loop being promoted by palmitoylation. We suggest that by recruiting residues 709–728 close to the membrane, palmitoylation of C739 facilitates engagement of this region with the XIP domain, which is structurally constrained because it is immediately adjacent to TM5 at the N-terminal end of the f-loop. However, since a soluble biotinylated XIP peptide affinity purifies unpalmitoylated NCX1 less efficiently than WT, the impact of palmitoylation must also be to restructure the f-loop to create a binding site for XIP in addition to bringing this part of the protein closer to the membrane.

The XIP docking site we have identified lies only 15 amino acids on the C-terminal side of CBD2. As the functional consequence of Ca binding to CBD2 is the relief of NCX1 inactivation, our investigation suggests a scenario in which engagement of XIP with its docking domain destabilizes the Ca binding sites in CBD2 to promote NCX1 inactivation. Considering the transition from inactivated to activated state, Ca binding to CBD2 may reverse XIP binding and/or efficacy. As well as being important for binding to 709–728, the polybasic nature of XIP may also be important to disturb the co-ordination of Ca ions by acidic residues in CBD2. Although we have previously reported Ca activation of NCX1 is not changed by palmitoylation, we used Ca concentrations that likely influence only the high-affinity binding site CBD1 (Reilly et al., 2015). An important prediction arising from this study that will be addressed in future experiments is that by modifying the ability of XIP to engage its docking site, palmitoylation indirectly regulates the Ca affinity of CBD2 in full-length NCX1.

How does NCX1 palmitoylation ultimately control intracellular Ca? Our data indicate that NCX1-mediated transmembrane Ca fluxes are reduced when NCX1 is palmitoylated and enhanced when NCX1 is depalmitoylated, which implies that XIP tunes

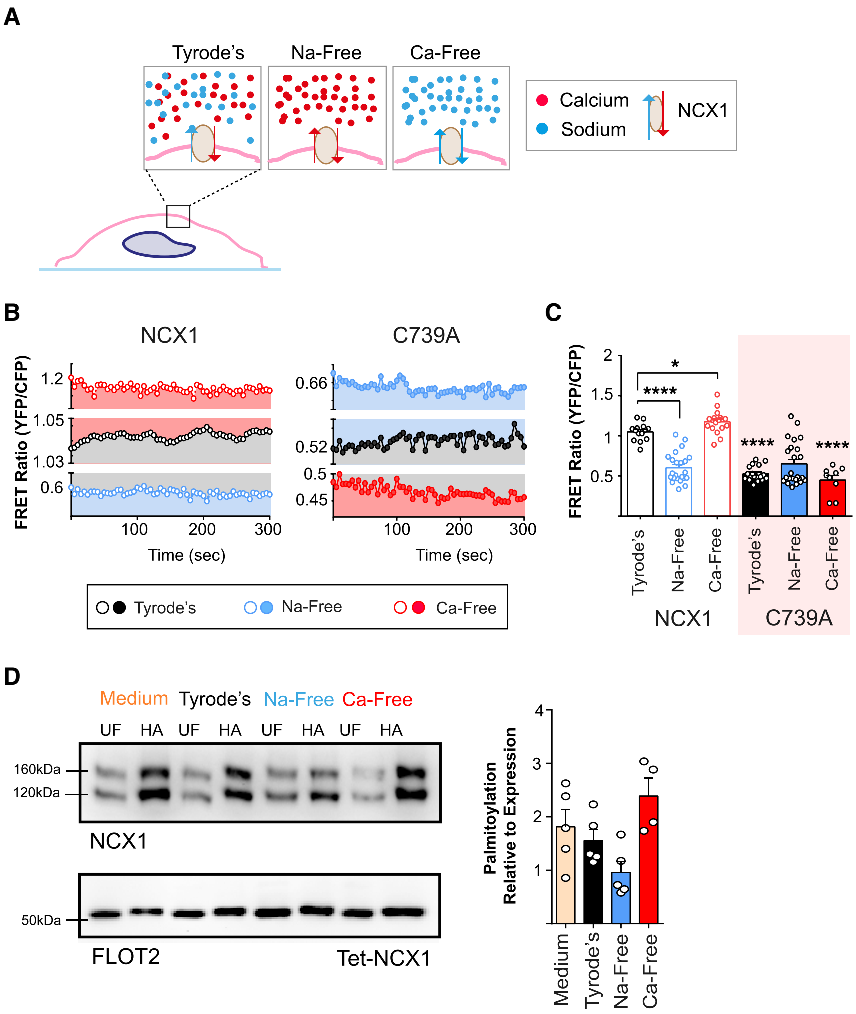

Figure 5. NCX1 Palmitoylation and the Extracellular Environment

(A) Schematic of the experimental intervention. HEK293-expressing NCX1 FRET sensors were incubated in Tyrode's buffer (containing physiological concentrations of Na and Ca) or modified Tyrode's buffer that was free of either Na (replaced with NMDG) or Ca (chelated with 1 mM EGTA).

(B) An example of NCX1-NCX1 FRET measurements in transiently transfected HEK293 cells expressing WT NCX1 (left, open circles) or unpalmitoylatable NCX1 (right, closed circles) in physiological (black), sodium-free (blue) and calcium-free (red) conditions.

(C) Sodium-free (NaF) solutions reduce, and calcium-free (CaF) solutions enhance, NCX1-NCX1 FRET, but only when NCX1 is palmitoylatable. ****$p < 0.0001$, *$p = 0.01$, calculated by unpaired t test. Statistical comparisons in C739A groups are to corresponding conditions for WT NCX1. N = 14 (WT, Tyrode's), 23 (WT, Na-free), 18 (WT, Ca-free), 19 (C739A Tyrode's), 24 (C739A Na-free), 9 (C739A, Ca-free).

(D) The same Na-free solutions reduce, and Ca-free solutions enhance, NCX1 palmitoylation. UF, unfractionated lysate; HA, purified palmitoylated fraction. The bar chart (right) shows NCX1 palmitoylation (HA fraction) normalized to expression (UF) following the indicated treatments.

NCX1 activity at rest and not simply following PIP2 mobilization. NCX1 inactivation is demonstrated to have a physiological role in the control of intracellular Ca by our experiments. Clearly NCX1 palmitoylation modifies XIP binding, but destabilization of Ca binding to CBD2 by XIP does not fully explain the NCX1 inactivation process. Although allosteric regulation by Na, Ca, and XIP are lost, the proteolytic removal of the entire NCX1 f-loop does not abolish exchanger activity, so CBD1, CBD2, and XIP are not required for ion transport (Hilgemann, 1990; Philipson et al., 1988). What is required for transporter activity, however, is the entry of ions into their transmembrane binding sites along extracellular and cytosolic vestibules. The complete cessation of transporter activity that occurs when XIP binds to its docking site could be explained if XIP binding prevented these ions from accessing the mouth of a cytosolic vestibule. This will be the subject of future experiments.

Previous experiments that identified residues 562–679 as essential for the functional effect of XIP (Maack et al., 2005) are not inconsistent with our finding that XIP binds elsewhere in the f-loop. Indeed, the fact that a functionally inactive XIP peptide affinity purified WT NCX1 equally as well as WT XIP suggests that XIP binding to and inactivation of NCX1 occur independently. The important implications of this observation are that experimental and therapeutic strategies to inhibit XIP activity must target a different region of the f-loop than those aiming to mimic XIP activity. The region of the f-loop required for XIP efficacy is likely smaller than has been defined to date, but it was beyond the scope of the current investigation to fine-map its location.

The fact that we were able to achieve modest co-purification of unpalmitoylatable NCX1 with XIP suggests that a low-affinity interaction with XIP is maintained when NCX1 is not palmitoylated. In keeping with this, exogenous XIP slightly increases the FRET activity of unpalmitoylatable NCX1, implying that it retains some ability to regulate unpalmitoylated NCX1. This is consistent with our earlier finding that NCX1 shows reduced (but not entirely absent) inactivation when unpalmitoylated (Reilly et al., 2015) and implies that even when binding of XIP to its docking site is reduced (when NCX1 is not palmitoylated), a functional effect on NCX1 can be achieved.

NCX1-NCX1 FRET

It has previously been shown in *Xenopus* plasma membrane sheets that NCX1-NCX1 FRET increases dynamically as Ca occupies the CBDs (John et al., 2011). Multiple structural rearrangements leading to altered NCX1-NCX1 FRET are evidently possible, some of which are independent of palmitoylation. Palmitoylation-induced changes in NCX1-NCX1 FRET only occur

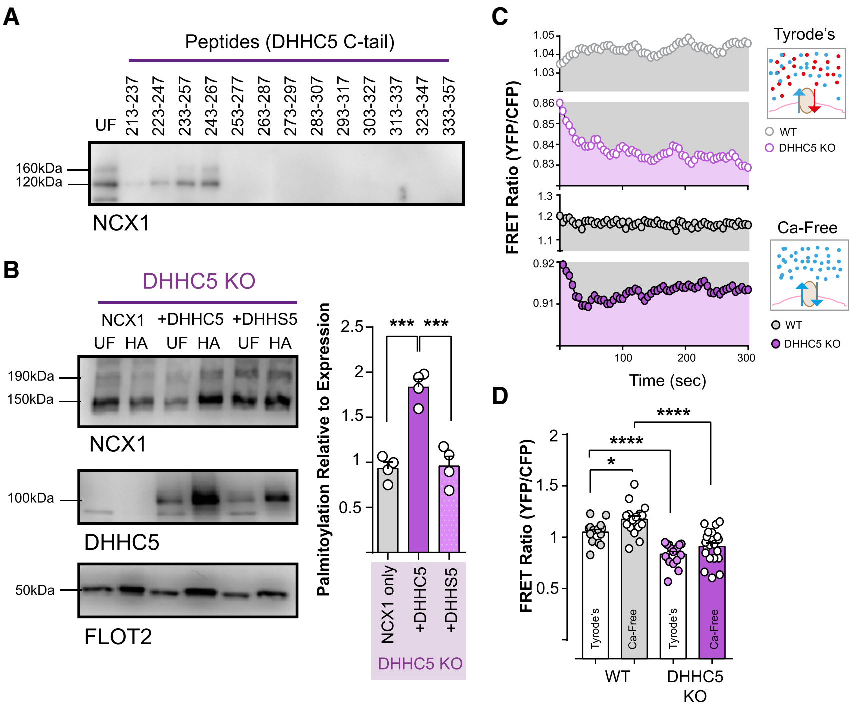

Figure 6. NCX1 Relationship with zDHHC5

(A) An array of biotinylated zDHHC5 peptides representing the zDHHC5 C tail affinity purifies NCX1 from rat ventricular lysates.

(B) NCX1 palmitoylation is increased by co-expression of WT, but not catalytically inactive zDHHC5, in zDHHC5 KO cells. ***p = 0.0002 (knockout [KO] versus +zDHHC5), 0.0009 (+DHHC5 versus zDHHS5), calculated by unpaired t test, N = 4.

(C) NCX1-NCX1 FRET recordings in HEK and zDHHC5 KO cells.

(D) NCX1-NCX1 FRET is reduced in zDHHC5 KO cells and is no longer sensitive to changes in extracellular ion concentrations. ****p < 0.0001, *p = 0.01, calculated by unpaired t test. N = 14 (WT, Tyrode's), 18 (WT, Ca-free), 16 (DHHC5 KO Tyrode's), and 20 (DHHC5 KO, Ca-free),

when lipid rafts can form (discussed below), which suggests that there is a requirement for particular phospholipid(s) and/or the membrane's physical properties to support palmitoylation-induced restructuring of the NCX1 dimer. We propose that the "palmitoylated-high FRET form" of NCX1 identified in this investigation represents a species that can inactivate, while the "unpalmitoylated-low FRET form" is less capable of inactivating, because it has a lower affinity for XIP. Our finding that Ca-free conditions enhance both NCX1 palmitoylation and FRET are important, because this suggests that Na binding to its allosteric regulatory site, already established to promote NCX1 inactivation, may trigger a positive feedback loop to inactivate NCX1 by enhancing its palmitoylation, sensitizing NCX1 XIP. Although the location of the allosteric regulatory site for Na is as-yet unknown, it does not lie within the CBDs (Boyman et al., 2009, 2011). The possibility that this site is close to the palmitoylation site in NCX1 should now be considered.

Dynamic NCX1 Palmitoylation at the Cell Surface

We have previously identified the Golgi as the principal site of NCX1 palmitoylation, because a YFP fusion protein that includes the NCX1 palmitoylation site (but not the transmembrane domains) becomes trapped in the Golgi apparatus (Plain et al., 2017; Reilly et al., 2015). Here, we extend these findings and report that following its delivery to the cell surface, NCX1 is dynamically depalmitoylated and repalmitoylated by APT1 and zDHHC5, respectively. The localization of a palmitoylated NCX1 f-loop to the Golgi suggests that the Golgi-localized NCX1 zDHHC-PAT is a higher-capacity palmitoylation system than zDHHC5, which is consistent with the Golgi being a major cellular "hub" of protein palmitoylation (Ernst et al., 2018; Salaun et al., 2010). Alternatively, palmitoylation of NCX1 by zDHHC5 may require the NCX1 transmembrane domains to be present. Regardless of this, however, our observations suggest that following its delivery to the cell surface, cellular mechanisms exist to dynamically modify the sensitivity of NCX1 to inactivation via changes to its palmitoylation status, as brought about by APT1 and zDHHC5. Substrate "recognition rules" for thioesterase enzymes remain largely uninvestigated. Our experiments here show that the amphipathic α-helix that is required for NCX1 palmitoylation is also recognized by APT1. Recruitment of zDHHC-PATs and thioesterases to NCX1 may not, therefore, be independent events.

Little information exists about the regulatory events that control substrate palmitoylation and depalmitoylation, although palmitoylation cascades (Abrami et al., 2017) and phosphorylation-dependent substrate palmitoylation (Brigidi et al., 2015) have both been reported in recent years. In this study, we have identified one cellular event that regulates NCX1 palmitoylation by zDHHC5, ion binding site occupancy, which restructures the NCX1 f-loop to alter the ability of zDHHC5 to palmitoylate NCX1. It is likely that other mechanisms that remain to be identified regulate NCX1 palmitoylation at the cell surface. Indeed, our finding that NCX1 palmitoylation is not entirely abolished in zDHHC5 KO cells implies that other zDHHC-PATs, possibly both in the secretory pathway and at the cell surface, also regulate NCX1.

Palmitoylation and the Microdomain Localization of NCX1

This investigation is the first report that demonstrates that a multi-pass membrane domain protein can have significant affinity for the ordered phase of phase-separated GPMVs. The concept that lipid-lipid interactions lead to the dynamic formation of lipid rafts in live cells (Simons and Ikonen, 1997) is not universally accepted (Douglass and Vale, 2005; Munro, 2003). This investigation provides direct support for the concept that such rafts exist in intact cells. This is because palmitoylation modifies the FRET behavior of NCX1 dimers only when rafts are able to

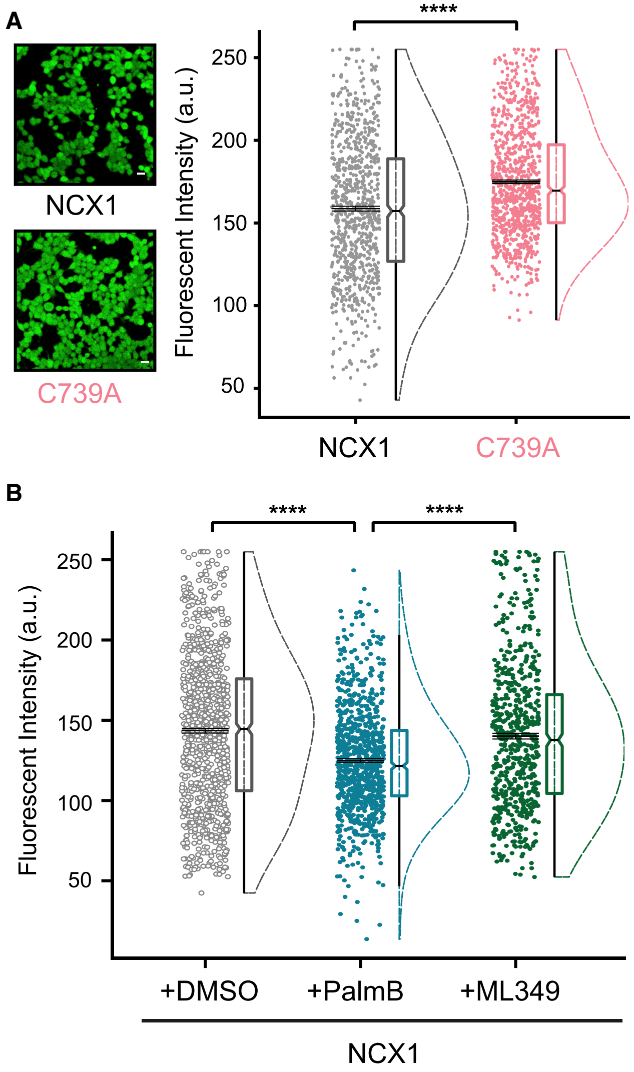

Figure 7. Impact of NCX1 Palmitoylation on Intracellular Ca

(A) Steady-state intracellular Ca was evaluated using the fluorescent indicator Fluo 4 based on measured fluorescence intensity in the cells. Fluorescence intensity in FT-293 cells expressing WT NCX1 is lower than in FT-293 cells expressing unpalmitoylatable NCX1 (C739A) (WT: 158.69 ± 1.44, C739A: 169.64 ± 1.10). Data in each panel are presented as scattered individual data points with standard error, boxplot with median and half violin plot. ****p < 0.0001, calculated by unpaired t test. N = 855 (WT NCX1) and 915 (C739A). Scale bar, 20 μm.

(B) Elevating NCX1 palmitoylation with the thioesterase inhibitor PalmB reduced intracellular Ca signals in cells expressing WT NCX1, whereas the APT2 inhibitor ML349 is without effect (DMSO, 143.51 ± 1.49; PalmB, 124.98 ± 1.11; ML349, 140.12 ± 1.79). ****p < 0.0001, calculated by unpaired t test. N = 991 (DMSO), 901 (PalmB), and 642 (ML349).

form. Interventions that prevented rafts from forming reduced the FRET activity of WT NCX1 to match that of unpalmitoylatable NCX1, whereas the FRET activity of unpalmitoylatable NCX1 was insensitive to the manipulation of the membrane. The cholesterol-dependent and independent clustering of peripheral (Zhou and Hancock, 2015) and single-pass integral (Lorent et al., 2017) membrane proteins into microdomains of unique lipid composition is well established. The palmitoylation of H-Ras, for example, controls its affinity for lipid ordered domains and enhances H-Ras clustering in these domains (Janosi et al., 2012). We propose that a similar relationship exists for NCX1; palmitoylation changes the affinity of NCX1 for a microdomain in the surface membrane and promotes NCX1-NCX1 FRET in this microdomain.

Our results show that the presence of WT, but not unpalmitoylatable, NCX1 impaired the phase separation of GPMVs. The simplest explanation for this phenomenon is that the temperature at which phase separation occurs is decreased by the presence of WT (but not unpalmitoylatable) NCX1. This impact of WT NCX1 on GPMV phase separation suggests that the presence of a palmitoylated protein can significantly alter the behavior of the lipids with which it interacts and is another example of the dynamic palmitoylation of an integral membrane protein profoundly changing the behavior of its phospholipid environment (Fuller et al., 2016; Hilgemann et al., 2013, 2019; Reilly et al., 2015). We suggest that the ability of NCX1 to bind PIP2 via its XIP domain establishes a scenario in which the attraction of palmitoylated NCX1 to lipid-ordered domains “conflicts” with the binding of PIP2 elsewhere in the protein. This is strikingly similar to the behavior of palmitoylated and farnesylated H-Ras in molecular dynamics simulations. The presence of saturated palmitate attracts H-Ras to the lipid ordered phase, and the presence of unsaturated farnesyl attracts it to the disordered phase, resulting in the clustering of such dually lipidated proteins at order/disorder boundaries (Janosi et al., 2012).

Concluding Remarks

We identify a small region of the NCX1 f-loop that is required for XIP binding that is distinct from the region of the f-loop required for XIP function. We report that the dynamic regulation of NCX1 palmitoylation at the cell surface controls its ability to inactivate (by modifying the interaction between XIP and its binding site) and affinity for lipid microdomains. The modified XIP sensitivity ultimately facilitates palmitoylation-dependent control of NCX1-mediated transmembrane Ca flux and hence cytosolic Ca levels. Given the importance of inactivation in controlling NCX1-mediated Ca influx, future research to identify the pathways that control NCX1 palmitoylation could enable the inactivation of this ubiquitous ion transporter to be manipulated.

STAR★METHODS

Detailed methods are provided in the online version of this paper and include the following:

- KEY RESOURCES TABLE
- RESOURCE AVAILABILITY
  - Lead Contact
  - Materials Availability
  - Data and Code Availability
- EXPERIMENTAL MODEL AND SUBJECT DETAILS
  - Ethics
  - Immortalized Cell Lines and Plasmids
  - Generation of zDHHC5 knockout cells
  - Ventricular Myocytes and Cardiac Tissue

- METHOD DETAILS
  - Drugs and Reagents
  - Purification of palmitoylated proteins
  - Protein Cross-linking
  - FRET Imaging
  - Giant Plasma Membrane Vesicles (GPMV)
  - Manipulating Cholesterol in Intact Cells
  - Peptide Affinity Purification and LC-MS/MS
  - Intracellular Calcium Imaging
- QUANTIFICATION AND STATISTICAL ANALYSIS

SUPPLEMENTAL INFORMATION

Supplemental Information can be found online at https://doi.org/10.1016/j.celrep.2020.107697.

ACKNOWLEDGMENTS

We acknowledge financial support from the British Heart Foundation (FS/14/68/30988 to W.F. and N.J.F. and SP/16/3/32317 to W.F.) and Centre of Research Excellence award RE/18/6/34217.

AUTHOR CONTRIBUTIONS

Conceptualization, C.G. and W.F.; Methodology, C.G.; Investigation: C.G., F.P., and N.J.F.; Resources, A.D.R., J.H., and G.S.B.; Writing – Original Draft, W.F.; Writing – Review & Editing, C.G. and N.J.F.; Supervision, W.F.; Project Administration, W.F.; Funding Acquisition, N.J.F. and W.F.

DECLARATION OF INTERESTS

The authors declare no competing interests.

Received: January 3, 2020
Revised: April 7, 2020
Accepted: May 6, 2020
Published: June 9, 2020

REFERENCES

Abrami, L., Dallavilla, T., Sandoz, P.A., Demir, M., Kunz, B., Savoglidis, G., Hatzimanikatis, V., and van der Goot, F.G. (2017). Identification and dynamics of the human ZDHHC16-ZDHHC6 palmitoylation cascade. eLife *6*, e27826.

Adibekian, A., Martin, B.R., Chang, J.W., Hsu, K.L., Tsuboi, K., Bachovchin, D.A., Speers, A.E., Brown, S.J., Spicer, T., Fernandez-Vega, V., et al. (2012). Confirming target engagement for reversible inhibitors in vivo by kinetically tuned activity-based probes. J. Am. Chem. Soc. *134*, 10345–10348.

Allen, M., Poggiali, D., Whitaker, K., Marshall, T.R., and Kievit, R.A. (2019). Raincloud plots: a multi-platform tool for robust data visualization. Wellcome Open Res. *4*, 63.

Boyman, L., Mikhasenko, H., Hiller, R., and Khananshvili, D. (2009). Kinetic and equilibrium properties of regulatory calcium sensors of NCX1 protein. J. Biol. Chem. *284*, 6185–6193.

Boyman, L., Hagen, B.M., Giladi, M., Hiller, R., Lederer, W.J., and Khananshvili, D. (2011). Proton-sensing Ca2+ binding domains regulate the cardiac Na+/Ca2+ exchanger. J. Biol. Chem. *286*, 28811–28820.

Brigidi, G.S., Santyr, B., Shimell, J., Jovellar, B., and Bamji, S.X. (2015). Activity-regulated trafficking of the palmitoyl-acyl transferase DHHC5. Nat. Commun. *6*, 8200.

Chlanda, P., Mekhedov, E., Waters, H., Sodt, A., Schwartz, C., Nair, V., Blank, P.S., and Zimmerberg, J. (2017). Palmitoylation contributes to membrane curvature in influenza A virus assembly and hemagglutinin-mediated membrane fusion. J. Virol. *91*, 91.

Dai, W., Laforest, B., Tyan, L., Shen, K.M., Nadadur, R.D., Alvarado, F.J., Mazurek, S.R., Lazarevic, S., Gadek, M., Wang, Y., et al. (2019). A calcium transport mechanism for atrial fibrillation in Tbx5-mutant mice. eLife *8*, e41814.

Diaz-Rohrer, B.B., Levental, K.R., Simons, K., and Levental, I. (2014). Membrane raft association is a determinant of plasma membrane localization. Proc. Natl. Acad. Sci. USA *111*, 8500–8505.

Douglass, A.D., and Vale, R.D. (2005). Single-molecule microscopy reveals plasma membrane microdomains created by protein-protein networks that exclude or trap signaling molecules in T cells. Cell *121*, 937–950.

Ernst, A.M., Syed, S.A., Zaki, O., Bottanelli, F., Zheng, H., Hacke, M., Xi, Z., Rivera-Molina, F., Graham, M., Rebane, A.A., et al. (2018). S-palmitoylation sorts membrane cargo for anterograde transport in the Golgi. Dev. Cell *47*, 479–493.e477.

Eshcol, J.O., Harding, A.M., Hattori, T., Costa, V., Welsh, M.J., and Benson, C.J. (2008). Acid-sensing ion channel 3 (ASIC3) cell surface expression is modulated by PSD-95 within lipid rafts. Am. J. Physiol. Cell Physiol. *295*, C732–C739.

Flesch, M., Schwinger, R.H., Schiffer, F., Frank, K., Südkamp, M., Kuhn-Regnier, F., Arnold, G., and Böhm, M. (1996). Evidence for functional relevance of an enhanced expression of the Na(+)-Ca2+ exchanger in failing human myocardium. Circulation *94*, 992–1002.

Fuller, W., Reilly, L., and Hilgemann, D.W. (2016). S-palmitoylation and the regulation of NCX1. Channels (Austin) *10*, 75–77.

Gök, C., and Fuller, W. (2020). Regulation of NCX1 by palmitoylation. Cell Calcium *86*, 102158.

Hilgemann, D.W. (1990). Regulation and deregulation of cardiac Na(+)-Ca2+ exchange in giant excised sarcolemmal membrane patches. Nature *344*, 242–245.

Hilgemann, D.W., Matsuoka, S., Nagel, G.A., and Collins, A. (1992). Steady-state and dynamic properties of cardiac sodium-calcium exchange. Sodium-dependent inactivation. J. Gen. Physiol. *100*, 905–932.

Hilgemann, D.W., Yaradanakul, A., Wang, Y., and Fuster, D. (2006). Molecular control of cardiac sodium homeostasis in health and disease. J. Cardiovasc. Electrophysiol. *17* (*Suppl 1*), S47–S56.

Hilgemann, D.W., Fine, M., Linder, M.E., Jennings, B.C., and Lin, M.J. (2013). Massive endocytosis triggered by surface membrane palmitoylation under mitochondrial control in BHK fibroblasts. eLife *2*, e01293.

Hilgemann, D.W., Dai, G., Collins, A., Lariccia, V., Magi, S., Deisl, C., and Fine, M. (2018). Lipid signaling to membrane proteins: From second messengers to membrane domains and adapter-free endocytosis. J. Gen. Physiol. *150*, 211–224.

Hilgemann, D.W., Lin, M.J., Fine, M., and Deisl, C. (2019). On the existence of endocytosis driven by membrane phase separations. Biochim. Biophys. Acta Biomembr. *1862*, 183007.

Howie, J., Tulloch, L.B., Shattock, M.J., and Fuller, W. (2013). Regulation of the cardiac Na(+) pump by palmitoylation of its catalytic and regulatory subunits. Biochem. Soc. Trans. *41*, 95–100.

Howie, J., Reilly, L., Fraser, N.J., Vlachaki Walker, J.M., Wypijewski, K.J., Ashford, M.L., Calaghan, S.C., McClafferty, H., Tian, L., Shipston, M.J., et al. (2014). Substrate recognition by the cell surface palmitoyl transferase DHHC5. Proc. Natl. Acad. Sci. USA *111*, 17534–17539.

Janosi, L., Li, Z., Hancock, J.F., and Gorfe, A.A. (2012). Organization, dynamics, and segregation of Ras nanoclusters in membrane domains. Proc. Natl. Acad. Sci. USA *109*, 8097–8102.

John, S.A., Ribalet, B., Weiss, J.N., Philipson, K.D., and Ottolia, M. (2011). Ca2+-dependent structural rearrangements within Na+-Ca2+ exchanger dimers. Proc. Natl. Acad. Sci. USA *108*, 1699–1704.

Kettlewell, S., Seidler, T., and Smith, G.L. (2009). The effects of over-expression of the FK506-binding protein FKBP12.6 on K(+) currents in adult rabbit ventricular myocytes. Pflugers Arch. *458*, 653–660.

Khananshvili, D. (2020). Basic and editing mechanisms underlying ion transport and regulation in NCX variants. Cell Calcium *85*, 102131.

Kirkton, R.D., and Bursac, N. (2011). Engineering biosynthetic excitable tissues from unexcitable cells for electrophysiological and cell therapy studies. Nat. Commun. *2*, 300.

Larsen, J.B., Jensen, M.B., Bhatia, V.K., Pedersen, S.L., Bjørnholm, T., Iversen, L., Uline, M., Szleifer, I., Jensen, K.J., Hatzakis, N.S., and Stamou, D. (2015). Membrane curvature enables N-Ras lipid anchor sorting to liquid-ordered membrane phases. Nat. Chem. Biol. *11*, 192–194.

Levental, I., Grzybek, M., and Simons, K. (2010). Greasing their way: lipid modifications determine protein association with membrane rafts. Biochemistry *49*, 6305–6316.

Liao, J., Li, H., Zeng, W., Sauer, D.B., Belmares, R., and Jiang, Y. (2012). Structural insight into the ion-exchange mechanism of the sodium/calcium exchanger. Science *335*, 686–690.

Liao, J., Marinelli, F., Lee, C., Huang, Y., Faraldo-Gómez, J.D., and Jiang, Y. (2016). Mechanism of extracellular ion exchange and binding-site occlusion in a sodium/calcium exchanger. Nat. Struct. Mol. Biol. *23*, 590–599.

Lin, D.T., and Conibear, E. (2015). ABHD17 proteins are novel protein depalmitoylases that regulate N-Ras palmitate turnover and subcellular localization. eLife *4*, e11306.

Linder, M.E., and Deschenes, R.J. (2007). Palmitoylation: policing protein stability and traffic. Nat. Rev. Mol. Cell Biol. *8*, 74–84.

Lorent, J.H., Diaz-Rohrer, B., Lin, X., Spring, K., Gorfe, A.A., Levental, K.R., and Levental, I. (2017). Structural determinants and functional consequences of protein affinity for membrane rafts. Nat. Commun. *8*, 1219.

Maack, C., Ganesan, A., Sidor, A., and O'Rourke, B. (2005). Cardiac sodium-calcium exchanger is regulated by allosteric calcium and exchanger inhibitory peptide at distinct sites. Circ. Res. *96*, 91–99.

Matsuoka, S., Nicoll, D.A., Reilly, R.F., Hilgemann, D.W., and Philipson, K.D. (1993). Initial localization of regulatory regions of the cardiac sarcolemmal Na(+)-Ca2+ exchanger. Proc. Natl. Acad. Sci. USA *90*, 3870–3874.

Matsuoka, S., Nicoll, D.A., He, Z., and Philipson, K.D. (1997). Regulation of cardiac Na(+)-Ca2+ exchanger by the endogenous XIP region. J. Gen. Physiol. *109*, 273–286.

Milletti, F. (2012). Cell-penetrating peptides: classes, origin, and current landscape. Drug Discov. Today *17*, 850–860.

Mitchell, D.A., Vasudevan, A., Linder, M.E., and Deschenes, R.J. (2006). Protein palmitoylation by a family of DHHC protein S-acyltransferases. J. Lipid Res. *47*, 1118–1127.

Munoz, I.M., Szyniarowski, P., Toth, R., Rouse, J., and Lachaud, C. (2014). Improved genome editing in human cell lines using the CRISPR method. PLoS ONE *9*, e109752.

Munro, S. (2003). Lipid rafts: elusive or illusive? Cell *115*, 377–388.

Ohtsuka, M., Takano, H., Suzuki, M., Zou, Y., Akazawa, H., Tamagawa, M., Wakimoto, K., Nakaya, H., and Komuro, I. (2004). Role of Na+-Ca2+ exchanger in myocardial ischemia/reperfusion injury: evaluation using a heterozygous Na+-Ca2+ exchanger knockout mouse model. Biochem. Biophys. Res. Commun. *314*, 849–853.

Ottolia, M., Nicoll, D.A., and Philipson, K.D. (2009). Roles of two Ca2+-binding domains in regulation of the cardiac Na+-Ca2+ exchanger. J. Biol. Chem. *284*, 32735–32741.

Ottolia, M., Torres, N., Bridge, J.H., Philipson, K.D., and Goldhaber, J.I. (2013). Na/Ca exchange and contraction of the heart. J. Mol. Cell. Cardiol. *61*, 28–33.

Pei, Z., Xiao, Y., Meng, J., Hudmon, A., and Cummins, T.R. (2016). Cardiac sodium channel palmitoylation regulates channel availability and myocyte excitability with implications for arrhythmia generation. Nat. Commun. *7*, 12035.

Philipson, K.D., Longoni, S., and Ward, R. (1988). Purification of the cardiac Na+-Ca2+ exchange protein. Biochim. Biophys. Acta *945*, 298–306.

Plain, F., Congreve, S.D., Yee, R.S.Z., Kennedy, J., Howie, J., Kuo, C.W., Fraser, N.J., and Fuller, W. (2017). An amphipathic α-helix directs palmitoylation of the large intracellular loop of the sodium/calcium exchanger. J. Biol. Chem. *292*, 10745–10752.

Qi, Y., Andolfi, L., Frattini, F., Mayer, F., Lazzarino, M., and Hu, J. (2015). Membrane stiffening by STOML3 facilitates mechanosensation in sensory neurons. Nat. Commun. *6*, 8512.

Reeves, J.P., and Condrescu, M. (2008). Ionic regulation of the cardiac sodium-calcium exchanger. Channels (Austin) *2*, 322–328.

Reilly, L., Howie, J., Wypijewski, K., Ashford, M.L., Hilgemann, D.W., and Fuller, W. (2015). Palmitoylation of the Na/Ca exchanger cytoplasmic loop controls its inactivation and internalization during stress signaling. FASEB J. *29*, 4532–4543.

Ren, X., Nicoll, D.A., Galang, G., and Philipson, K.D. (2008). Intermolecular cross-linking of Na+-Ca2+ exchanger proteins: evidence for dimer formation. Biochemistry *47*, 6081–6087.

Salaun, C., Greaves, J., and Chamberlain, L.H. (2010). The intracellular dynamic of protein palmitoylation. J. Cell Biol. *191*, 1229–1238.

Schindelin, J., Arganda-Carreras, I., Frise, E., Kaynig, V., Longair, M., Pietzsch, T., Preibisch, S., Rueden, C., Saalfeld, S., Schmid, B., et al. (2012). Fiji: an open-source platform for biological-image analysis. Nat. Methods *9*, 676–682.

Sezgin, E., Kaiser, H.J., Baumgart, T., Schwille, P., Simons, K., and Levental, I. (2012). Elucidating membrane structure and protein behavior using giant plasma membrane vesicles. Nat. Protoc. *7*, 1042–1051.

Shipston, M.J. (2011). Ion channel regulation by protein palmitoylation. J. Biol. Chem. *286*, 8709–8716.

Simons, K., and Ikonen, E. (1997). Functional rafts in cell membranes. Nature *387*, 569–572.

Voigt, N., Li, N., Wang, Q., Wang, W., Trafford, A.W., Abu-Taha, I., Sun, Q., Wieland, T., Ravens, U., Nattel, S., et al. (2012). Enhanced sarcoplasmic reticulum Ca2+ leak and increased Na+-Ca2+ exchanger function underlie delayed afterdepolarizations in patients with chronic atrial fibrillation. Circulation *125*, 2059–2070.

Wypijewski, K.J., Tinti, M., Chen, W., Lamont, D., Ashford, M.L., Calaghan, S.C., and Fuller, W. (2015). Identification of caveolar resident proteins in ventricular myocytes using a quantitative proteomic approach: dynamic changes in caveolar composition following adrenoceptor activation. Mol. Cell. Proteomics *14*, 596–608.

Zhou, Y., and Hancock, J.F. (2015). Ras nanoclusters: Versatile lipid-based signaling platforms. Biochim. Biophys. Acta *1853*, 841–849.

Zhou, Y., Maxwell, K.N., Sezgin, E., Lu, M., Liang, H., Hancock, J.F., Dial, E.J., Lichtenberger, L.M., and Levental, I. (2013). Bile acids modulate signaling by functional perturbation of plasma membrane domains. J. Biol. Chem. *288*, 35660–35670.

STAR★METHODS

KEY RESOURCES TABLE

REAGENT or RESOURCE	SOURCE	IDENTIFIER
Antibodies		
NCX1	SWANT	Cat#R3F1; RRID:AB_2716744
FLOT2	BD Biosciences	Cat#610384; RRID:AB_397767
DHHC5	Sigma	Cat#HPA014670; RRID:AB_2257442
GFP	Abcam	Cat#ab32146; RRID:AB_732717
Chemicals, Peptides, and Recombinant Proteins		
NCX1 peptide (NCX1$^{740-756}$) with N-terminal biotin	Alta Biosciences	N/A
DHHC5 peptides (C-tail: DHHC5$^{213-237,223-247,233-257,243-267,253-277,263-287,273-297,283-307,293-317,303-327,313-337,323-347,333-357}$) all with N-terminal biotin	Alta Biosciences	N/A
XIP (WT and K229Q XIP; NCX1$^{219-238}$) with N-terminal biotin	Alta Biosciences	N/A
PalmB	Calbiochem	Cat#178501
ML348	Dr. Brent Martin, University of Michigan	N/A
ML349	Dr. Brent Martin, University of Michigan	N/A
Filipin	Sigma	Cat#F9765
Ctx Alexa Fluor 647	Invitrogen	Cat#C34778
FAST-DiI	Invitrogen	Cat#D7756
Palmitic Acid	Sigma	Cat#0500
2-Bromopalmitate	Sigma	Cat#238422
Methyl-β-cyclodextrin	Sigma	Cat#332615
Critical Commercial Assays		
Fluo-4 Direct Calcium Imaging	Invitrogen	Cat#F10472
Experimental Models: Cell Lines		
FT-293	Invitrogen	Cat#R70007
HEK293T	ATCC	CRL-1573
Experimental Models: Organisms/Strains		
New Zealand White Rabbit	Envigo	444
Sprague Dawley Rat	Envigo	002
Oligonucleotides		
NCX1 Δ709-728 cloning, forward: 5′-GGACAAACGATGAA TGTGGAGAGGAGAAGC-3′	Eurofins Genomics	N/A
NCX1 Δ709-728 cloning, reverse: 5′-ATTCATCGTTTGTCC CAACCACAAGGGC-3′	Eurofins Genomics	N/A
C739A NCX1 mutagenesis, forward: 5′-TGCATCAC ATAATCGAAAGCGGAGGGCAGCTTCTCCTC-3′	Eurofins Genomics	N/A
C739A NCX1 mutagenesis, reverse: 5′-GAGGAGAAG CTGCCCTCCGCTTTCGATTATGTGATGCA-3	Eurofins Genomics	N/A
Recombinant DNA		
Canine NCX1.1 cDNA	Kenneth Philipson, UCLA	N/A
Canine NCX1.1 with CFP or YFP inserted at amino acid 266	Michela Ottolia, UCLA	N/A
YFP fusion proteins to the N terminus the NCX1.1 intracellular loop	Reilly et al., 2015; Plain et al., 2017	N/A
Software and Algorithms		
GraphPad	Graphpad Prism v6	https://graphpad.com
ImageJ / Fiji	Schindelin et al., 2012	https://imagej.nih.gov/ij/
The Discovery Series Quantity One 1-D Analysis Software Version 4.6.6, PC	Bio-Rad	LIT-70-9600-Q1-466PC

CellPress
OPEN ACCESS

RESOURCE AVAILABILITY

Lead Contact

Further information and requests for resources and reagents should be directed to and will be fulfilled by the Lead Contact, William Fuller (will.fuller@glasgow.ac.uk).

Materials Availability

Plasmids and cell lines generated in this study are available from the Lead Contact.

Data and Code Availability

The published article includes all datasets generated or analyzed during this study.

EXPERIMENTAL MODEL AND SUBJECT DETAILS

Ethics

This study utilized primary cells from rats and rabbits. All protocols involving animals were approved by the University of Glasgow Animal Welfare and Ethics Review Board. Rat cardiac and brain tissues were collected post-mortem after sacrificing animals using a method designated Schedule 1 by the Animals (Scientific Procedures) Act 1986. Rabbit hearts were excised from terminally anaesthetized, heparin-treated animals under the authority of a Project License granted by the UK Home Office.

Immortalized Cell Lines and Plasmids

HEK293 cells, HEK293 derived FT293 cell expressing tet-inducible wild-type (WT)-NCX1 and C739A-NCX1 and DHHC5 KO cells were used in this investigation. HEK293 derived FT293 cell expressing tet-inducible wild-type (WT)-NCX1 and C739A-NCX1 were generated using the Invitrogen Flip-In T-Rex System (Reilly et al., 2015).

NCX1 FRET sensors with either CFP or YFP inserted at position 266 (John et al., 2011) were a kind gift from Prof Michela Ottolia (UCLA, USA). Position 739 in the NCX1 f-loop of both YFP and CFP sensors was mutated from Cysteine to Alanine using the Quikchange Lightning Site-Directed Mutagenesis kit (Agilent). YFP fusions to the NCX1 f-loop are described elsewhere (Plain et al., 2017). YFP-NCX1 Δ709-728 was generated using InFusion (Takara) cloning. Plasmid constructs were transfected using Lipofectamine2000 (Invitrogen) for HEK293 cell and Lipofectamine LTX (Invitrogen) for neonatal myocytes, according to the manufacturer's instructions. Full details of all plasmids and the oligonucleotides used to generate them are provided in the Key Resources Table.

Generation of zDHHC5 knockout cells

We evaluated guide RNAs (gRNA) targeted against zDHHC5 provided by Horizon Discovery (Cambridge, UK). The selected gRNA targets 57,673,152 to 57,673,171 of *Homo sapiens* chromosome 11, and demonstrated a 46% targeting efficiency in a genome cleavage detection assay (ThermoFisher Scientific). gRNA was inserted into vector pEsgRNA into which a puromycin resistance cassette had been added (Munoz et al., 2014), which was transfected into FT-293 cells expressing tet-inducible cas9. After the induction of cas9 and following selection with puromycin (3μg/ml), cells were sorted to one cell per well in 96 well plates. Genomic DNA was isolated after clone expansion, amplified with primers directed against DHHC5 exon 2 (CCCATGTGCTTTCCTTCATT forward and CAGCCTGAGTGACAGAGCAA reverse), and sequenced. Seven out of 25 clones sequenced had no detectable wild-type DHHC5 alleles; the clone selected had an additional 388 bases inserted at position 57,673,154 of chromosome 11. Cas9 was removed from this clone by transfection with pOG44 Flp-Recombinase, and cells were selected and maintained in the presence of zeocin.

Ventricular Myocytes and Cardiac Tissue

Neonatal ventricular myocytes (NRVMs) were isolated from Wistar rat pups of mixed sex between postnatal day 1-4 using 0.45mg/ml Collagenase (Roche) and 1.25mg/ml Pancreatin (Sigma) in ADS buffer (106mM NaCl, 20mM HEPES, 800μM NaH_2PO_4, 5mM KCl, 400μM $MgSO_4$, 5mM Glucose; pH: 7.4) as digestion solution. The ventricles were cut into pieces and digested in a shaking water bath at 37°C. Cell suspension was collected every 20min and each time fresh digestion solution was added to the undigested tissue. Collected cells were plated in 10cm cell culture dishes for 2h to allow fibroblasts to attach to the bottom (pre-plating), then transferred to either EHS-Laminin coated glass coverslips to be used for FRET imaging or gelatin coated wellplates. Cells were incubated under standard cell culture conditions at 37°C and 5% CO2 in Day-1 Medium (Dulbecco's Modified Eagle Medium (DMEM6171, GIBCO), Medium199 (M-199, GIBCO), 10% Horse Serum, 5% NCS, 1% Glutamine and 1% Pen-Strep). After 24h incubation, the culture medium was replaced with Day-2 Medium containing low serum (5% Horse Serum and 0.5% NCS).

Calcium-tolerant adult rabbit ventricular myocytes were isolated from the left ventricular free wall of male New Zealand White rabbits (2.8-3.2kg, 14-19wks) following perfusion with collagenase in the Langendorff mode, as described previously (Kettlewell et al., 2009).

Cardiac tissues were collected from rats at gestational day 18 and adult male rats (250-300 g, 7-9wks). Tissues were snap frozen with dry ice after harvesting.

METHOD DETAILS

Drugs and Reagents

APT inhibitors Palmostatin B (PalmB), ML348 and ML349, Palmitic Acid (PA) and 2-Bromopalmitate (2BP) were investigated in this study. PalmB, PA and 2BP were purchased from Sigma. ML348 and ML349 were generously provided by Dr Brent Martin, University of Michigan, USA. All compounds were dissolved in DMSO and comparisons made to an appropriate vehicle control.

Purification of palmitoylated proteins

Palmitoylated proteins were purified by resin-assisted capture of acylated proteins (acyl-RAC) following blockade of free cysteines with MMTS and cleavage of thioester bonds with neutral hydroxylamine, as described in detail elsewhere (Plain et al., 2017). Palmitoylation of NCX1 is expressed as the amount purified by the acyl-RAC reaction relative to its abundance in the corresponding unfractionated cell lysate.

Protein Cross-linking

NCX1 cross-linking was carried out using the cysteine-reactive homobifunctional crosslinker bismaleimidohexane (BMH). Cross-linking buffer (with final concentration 0.1mM BMH) was 10mM Tris-HCl at pH: 7.2 and 150mM NaCl. FT-293 cells expressing tetracycline-inducible NCX1 were plated on poly-l-lysine (PLL, Sigma) coated 6 well plates. Following 3 washes with cross-linking buffer without BMH, cells were treated with 0.1mM BMH for 5min at 37°C. After cross-linking, cells were washed 3 times and proteins solubilized for 30min at 4°C in lysis buffer with 1% Triton X-100 and protease inhibitors. Solubilized cell lysate were centrifuged at 17500 g for 5min to eliminate insoluble material before analyzing by SDS-PAGE.

FRET Imaging

FRET experiments were performed on NRVMs and HEK293 cells 24-48h after transfection with NCX1 FRET sensors. Cells were maintained in Tyrode's buffer (120mM NaCl, 5mM KCl, 1mM $MgCl_2$, 1.8mM $CaCl_2$ and 10mM HEPES; pH:7.4) at room temperature while FRET signals were recorded, unless an experiment is indicated as being either Calcium-free (CaF: 120mM NaCl, 5mM KCl, 1mM $MgCl_2$, 1mM EGTA and 10mM HEPES; pH:7.4) or Sodium-free (NaF: 120mM $NMDG^+$, 5mM KCl, 1mM $MgCl_2$, 1.8mM $CaCl_2$ and 10mM HEPES; pH:7.4). FRET activity was imaged by an inverted camera; Olympus 1X71, with PlanApon, 60X, NA 1.42 oil immersion objective, 0.17/FN 26.5 (Olympus, UK). The microscope was equipped with a CCD camera (cool SNAP HQ Monochrome, Photometrics) and a beam splitter optical device (Dual-channel simultaneous imaging system, DV^2 mag biosystem (ET-04-EM). MetaFluor 7.1 (Meta imaging system) was used for image acquisition and analysis. FRET ratio was measured as the changes in the background subtracted 480/545nm fluorescent emission intensity on excitation at 430nm.

Giant Plasma Membrane Vesicles (GPMV)

GPMVs were formed and analyzed as described previously (Sezgin et al., 2012). Briefly, HEK293 cells were plated onto poly-L-lysine (PLL, Sigma) coated 35mm dishes and transfected with CFP-tagged NCX1. After 24h, cells were washed twice with GPMV buffer (150mM NaCl, 2mM $CaCl_2$ and 10mM HEPES, pH: 7.4). GPMVs were formed using vesiculation buffer (25mM PFA, 2mM DTT, 0.001% Deoxycholate in GPMV buffer) for 1h at 37°C. The presence of DTT in the vesiculation buffer did not alter the palmitoylation status of NCX1 or flotillin 2 (Figure S1). The vesiculation buffer was supplemented with deoxycholate to improve phase separation (Zhou et al., 2013). Vesicles were visualized as free-floating dark spheres at the plane of the cells at 20X magnification. GPMV-rich cellular supernatant was harvested and transferred to centrifuge tubes and left to settle. GPMVs were labeled using FAST-DIL (5μg/ml, Invitrogen-D7756) for non-raft phase and CTxB-AlexaFluor647 (5μg/ml, Invitrogen-C34778) for raft phase, and imaged on a customised imaging chamber. GPMVs were imaged using a Zeiss LSM880 with Airyscan confocal microscopy. Diode (405-430nm), Argon (458nm, 488nm, 514nm) and HeNe633 (633nm) lasers were used for the excitation of blue (NCX1-CFP), green (FAST-DIL) and red (AlexaFluor647). The colocalization of NCX1 with lipid phases was analyzed using Coloc2 macro in Fiji (Schindelin et al., 2012).

Manipulating Cholesterol in Intact Cells

The manipulation of cholesterol in cells was achieved by using either MβCD (Sigma) to deplete cholesterol or SDS (Sigma) to force microdomain formation. Cholesterol was depleted as described previously (Eshcol et al., 2008; Qi et al., 2015): HEK293 cells were washed three times with PBS, then incubated with 5mM MβCD for 30min at 37°C. HEK293 cells were treated with 25μM SDS at 37°C for 15min to facilitate/increase microdomain formation. Following either MβCD or SDS incubation, cholesterol was detected with Filipin (50μg/ml in PBS, Sigma) as described (Qi et al., 2015) after fixation with 4% PFA for 10min at room temperature.

Peptide Affinity Purification and LC-MS/MS

Peptides with N-terminal biotin tags were synthesized by Alta Bioscience (Birmingham, UK). For affinity purification reactions, 20-50mg frozen powdered rat brain or heart tissue was lysed in 500μl lysis buffer (2mg/ml C12E10 in PBS supplemented with protease

inhibitors). Following solubilisation at 4°C for 30 min, insoluble material was removed by centrifuging at 17500 g for 5 min. Supernatants were applied to pre-equilibrated Streptavidin-Sepharose beads to preclear for 1h at 4°C, then incubated with 3μM biotinylated peptides overnight at 4°C. The next day, pre-equilibrated Streptavidin-Sepharose beads were added to the lysate-peptide mix and incubated for 3h at 4°C. The beads were washed 5 times with lysis buffer and interacting proteins were eluted in one of two ways. For the LC-MS/MS identification of NCX1-interacting proteins, beads were incubated with lysis buffer supplemented with 10μM $NCX1^{740-756}$ peptide and 1mM biotin for 1h at 4°C. After elution, the beads were washed once more with lysis buffer and the remaining proteins eluted with 2X LDS PAGE buffer supplemented with 100mM DTT. NCX1 interacting proteins were visualized by SDS-PAGE, stained with Coomassie, and identified using LC-MS/MS with a LTQ Orbitrap Velos Pro (Thermo) at the FingerPrints proteomics facility, University of Dundee.

Proteins interacting with biotinylated zDHHC5 and XIP peptides were eluted using 2x SDS-PAGE loading buffer and analyzed by western blot. Sequences of the custom-made peptides are provided in the Key Resources Table.

Intracellular Calcium Imaging

Intracellular calcium levels were monitored using the fluorescent calcium indicator Fluo4-Direct (Invitrogen) according to manufacturer's instructions. Tet-inducible stable cell lines (wild-type; WT NCX1 and unpalmitoylatable; C739A) were cultured on poly-l-lysine (PLL, Sigma) coated 35mm glass bottom dishes (MatTek Life Sciences). 16-24h after inducing NCX1 expression with tetracycline (1μg/ml), cells were loaded with Fluo4 for 1h at 37°C. Calcium indicator loaded cells were imaged using a Zeiss LSM8800 with Airyscan confocal microscopy ($\lambda_{excitation}$ = 494 nm, $\lambda_{emission}$ = 520 nm).

QUANTIFICATION AND STATISTICAL ANALYSIS

All data are presented as mean ± standard error of the mean. Quantitative differences between groups were assessed using One-way ANOVA analysis followed by appropriate post hoc t tests using GraphPad Prism. Intracellular calcium imaging data was evaluated and presented using Raincloud plots (Allen et al., 2019). The values of p and N are provided in individual figure legends

Cell Reports, Volume *31*

Supplemental Information

Dynamic Palmitoylation of the Sodium-Calcium Exchanger Modulates Its Structure, Affinity for Lipid-Ordered Domains, and Inhibition by XIP

Caglar Gök, Fiona Plain, Alan D. Robertson, Jacqueline Howie, George S. Baillie, Niall J. Fraser, and William Fuller

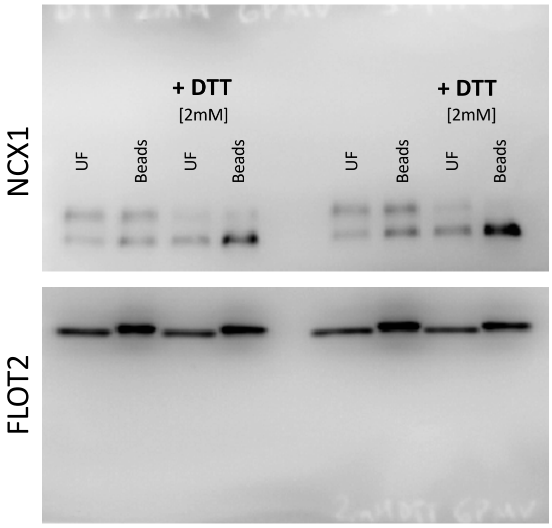

Supplementary Figure 1, Related to STAR Methods. The presence of DTT during GPMV preparation does not influence palmitoylation of NCX1 or flotillin 2. Palmitoylated proteins were prepared by resin-assisted capture of acylated proteins with or without pretreatment of lysates with 2mM DTT. The presence of DTT does not alter the quantity of NCX1 captured by the assay, but it does reduce intramolecular aggregation of NCX1 (the upper band on the western blot) during sample preparation. UF: unfractionated cell lysate, Beads: palmitoylated proteins.

Sequence of Peptides	
DHHC5 213-237	biotin-RGRTTNEQVTGKFRGGVNPFTNGCC
DHHC5 223-247	biotin-GKFRGGVNPFTNGCCNNVSRVLCSS
DHHC5 233-257	biotin-TNGCCNNVSRVLCSSPAPRYLGRPK
DHHC5 243-267	biotin-VLCSSPAPRYLGRPKKEKTIVIRPP
DHHC5 253-277	biotin-LGRPKKEKTIVIRPPFLRPEVSDGQ
DHHC5 263-287	biotin-VIRPPFLRPEVSDGQITVKIMDNGI
DHHC5 273-297	biotin-VSDGQITVKIMDNGIQGELRRTKSK
DHHC5 283-307	biotin-MDNGIQGELRRTKSKGSLEITESQS
DHHC5 293-317	biotin-RTKSKGSLEITESQSADAEPPPPPK
DHHC5 303-327	biotin-TESQSADAEPPPPPKPDLSRYTGLR
DHHC5 313-337	biotin-PPPPKPDLSRYTGLRTHLGLATNED
DHHC5 323-347	biotin-YTGLRTHLGLATNEDSSLLAKDSPP
DHHC5 333-357	biotin-ATNEDSSLLAKDSPPTPTMYKYRPG
NCX1 XIP with biotin	biotin-RRLLFYKYVYKRYRAGKQRG
NCX1 XIP without biotin	RRLLFYKYVYKRYRAGKQRG
NCX1 K229Q XIP	biotin-RRLLFYKYVYQRYRAGKQRG
NCX1 619-643	KYLFGQPVFRKVHAREHPILSTVIT
NCX1 629-653	KVHAREHPILSTVITIADEYDDKQP
NCX1 639-663	STVITIADEYDDKQPLTSKEEEERR
NCX1 649-673	DDKQPLTSKEEEERRIAEMGRPILG
NCX1 659-683	EEERRIAEMGRPILGEHTKLEVIIE
NCX1 669-693	RPILGEHTKLEVIIEESYEFKSTVD
NCX1 679-703	EVIIEESYEFKSTVDKLIKKTNLAL
NCX1 689-713	KSTVDKLIKKTNLALVVGTNSWREQ
NCX1 699-723	TNLALVVGTNSWREQFIEAITVSAG
NCX1 709-733	SWREQFIEAITVSAGEDDDDDECGE
NCX1 719-743	TVSAGEDDDDDECGEEKLPSCFDYV
NCX1 729-753	DECGEEKLPSCFDYVMHFLTVFWKV
NCX1 739-763	CFDYVMHFLTVFWKVLFAFVPPTEY
NCX1 742-766	YVMHFLTVFWKVLFAFVPPTEYWNG
NCX1 740-756	biotin-FDYVMHFLTVFWKVLFA

Supplemental Table 2, Related to STAR Methods. Sequences of peptides used in this investigation
